# China's fight to halt tree cover loss

**DOI:** 10.1098/rspb.2016.2559

**Published:** 2017-05-03

**Authors:** Antje Ahrends, Peter M. Hollingsworth, Philip Beckschäfer, Huafang Chen, Robert J. Zomer, Lubiao Zhang, Mingcheng Wang, Jianchu Xu

**Affiliations:** 1 Key Laboratory for Plant Diversity and Biogeography of East Asia, Kunming Institute of Botany, Chinese Academy of Sciences, Kunming 650201, People's Republic of China; 2 Royal Botanic Garden Edinburgh, 20A Inverleith Row, Edinburgh EH3 5LR, UK; 3 Chair of Forest Inventory and Remote Sensing, University of Göttingen, Büsgenweg 5, 37077 Göttingen, Germany; 4 World Agroforestry Centre, East and Central Asia, Kunming 650201, People's Republic of China; 5 Chinese Academy of Agricultural Sciences, 12 Zhongguancun Nan Dajie, CAAS Mailbox 195, Beijing 100081, People's Republic of China

**Keywords:** China, afforestation, deforestation, tree cover, biodiversity

## Abstract

China is investing immense resources for planting trees, totalling more than US$ 100 billion in the past decade alone. Every year, China reports more afforestation than the rest of the world combined. Here, we show that China's forest cover gains are highly definition-dependent. If the definition of ‘forest’ follows FAO criteria (including immature and temporarily unstocked areas), China has gained 434 000 km^2^ between 2000 and 2010. However, remotely detectable gains of vegetation that non-specialists would view as forest (tree cover higher than 5 m and minimum 50% crown cover) are an order of magnitude less (33 000 km^2^). Using high-resolution maps and environmental modelling, we estimate that approximately 50% of the world's forest with minimum 50% crown cover has been lost in the past approximately 10 000 years. China historically lost 1.9–2.7 million km^2^ (59–67%), and substantial losses continue. At the same time, most of China's afforestation investment targets environments that our model classes as unsuitable for trees. Here, gains detectable via satellite imagery are limited. Conversely, the regions where modest gains are detected are environmentally suitable but have received little afforestation investment due to conflicting land-use demands for agriculture and urbanization. This highlights the need for refined forest monitoring, and greater consideration of environmental suitability in afforestation programmes.

## 1. Background

China is home to one-fifth of the global population and is the most rapidly growing economy on Earth. Its GDP has increased by almost 270% since 2000 (constant prices). Measured by GDP Purchasing Power Parity, China is now the world's largest economy [1]. Yet, poverty reduction and food security remain fundamental challenges. China has the second largest number of poor in the world after India, with one-fifth of the population living on less than 2 US$ per day, and a GDP *per capita* that is only approximately 60% of the global average (in 2012) [2]. With more than 30% of the country prone to desertification, only a relatively small proportion of China's land is suitable for agriculture. Rapid urbanization and industrialization are competing with agriculture for land. Overall, the fast economic ascendance and competing interests for land result in great pressures on natural resources, including forests.

Following the devastating Yangtze River floods in 1988, China has undertaken tremendous efforts to reverse the trend of tree cover losses [3–6]. China's forestry expenditure per hectare is over three times higher than the global average, and has long exceeded that of the USA and Europe [7]. In the past decade alone, China invested more than US$ 100 billion into six key forestry programmes (see electronic supplementary material, Note S1). The aims of these programmes are to reduce environmental degradation, to create green spaces, to supply the enormous demand for forest products and to conserve biodiversity [5]. Their scale is globally unique. The ‘Three-North’ Shelterbelt Program [8] alone resulted in the planting of approximately 50 billion trees. Its aim is to build a 4500 km long wall of trees through the Gobi desert by 2050 to reduce sand storms. The Grain for Green Program [9,10] aims to convert crops to forests on steep slopes to reduce erosion and to increase the provision of forest products. With a total planned investment of US$ 40 billion and 40–60 million target households, it is regarded as the world's largest payment for ecosystem services scheme. The focus of China is thus on large-scale landscape manipulation and afforestation—often with single and sometimes exotic [8] species, which may not always be adapted to local conditions [3]. China now has the world's largest plantation area (approx. 800 000 km^2^ in 2010) [7]. At the same time, China is trying to reduce pressures on natural forests through strict bans on logging in primary forests and a massive expansion of its forest reserves to a current total of 2669 reserves covering 15% of the whole territory [11]. Thanks to these and other programmes, China has reportedly recovered from less than 13% forest cover in 1981 to more than 20% in 2010 [5]. China aims to expand its forest area by another 400 000 km^2^ (more than 25% relative to 2006 cover) between 2006 and 2020 [3].

However, forestry in China still faces substantial challenges. Forest area per person is only 25% of the global average and stocking volumes are low (78% of the global average), constituting only 2.9% of the world's total [12]. Demand for timber outstrips domestic production by more than 150 million m^3^ yr^−1^ [12]. China's industrial roundwood imports have increased by 192% between 2000 and 2013 alone [13], and account for more than one-third of the global, and almost two-thirds of all tropical wood imports. Partly to reduce pressures on scarce prime agricultural land, and partly to ameliorate degraded landscapes, forest plantations are often established on marginal land [5]. China has developed advanced aerial seeding technologies and immense efforts are made with irrigation [14]. However, several studies have cautioned that survival and growth rates of trees in marginal areas may be low [10,15]. One great challenge is the paucity of publicly available spatial data on afforestation in China with which to evaluate the efficacy of these measures [16].

A realistic understanding of tree-cover change in China is of global importance as China is responsible for a large part of the world's forests, it imports more timber than any other country on Earth [13] and invests more than all other countries combined in tree planting programmes. To evaluate this critically important topic, we use an environmental modelling approach combined with two recently published high-resolution global datasets on tree cover [17,18] to evaluate tree cover changes globally, and in China. Specifically, we assess (i) where the largest losses of tree cover have occurred and what the macro-scale economic correlates of countries that lose a lot of tree cover are; (ii) how China with its massive investment into afforestation fits into that picture; and (iii) whether that investment has led to effective returns in terms of tree cover gains.

**Figure 1.**
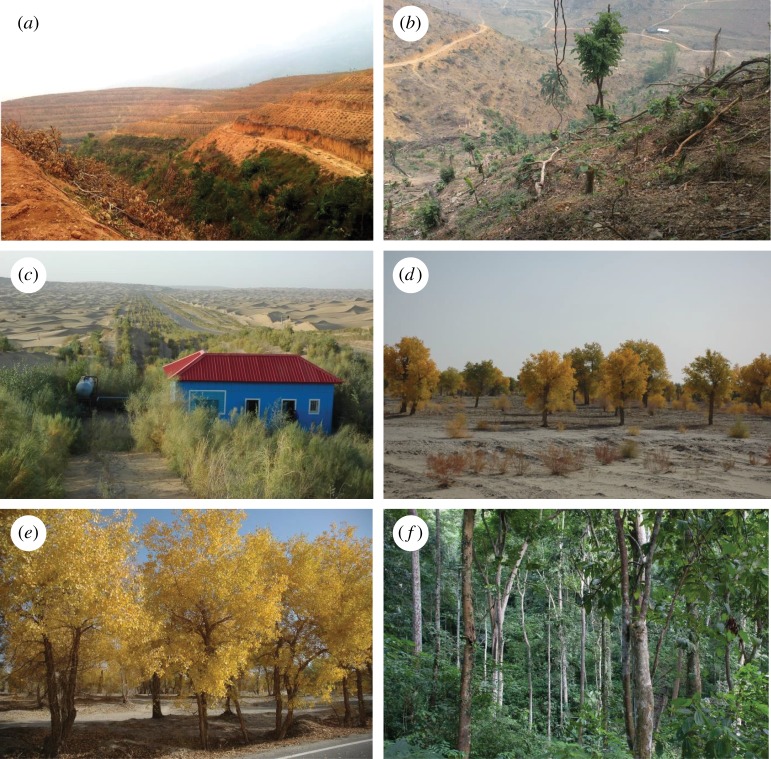
Different types of vegetation, which can all be classified as forest. (*a,b*) Land-use-based definitions also include unstocked land, which is designated for forest use. Here: clearing for rubber plantations. (*c*) Special purpose shrub planting in marginal areas. (*d*) Trees with crown cover of more than 10% and less than 20%. (*e*) Trees with crown cover of more than 20% and less than 50%. (*f*) Trees with crown cover of more than 50%.

A critical point in addressing this issue is the definition of what constitutes a ‘forest’. Forest can mean many different things to different people [19,20] (figure 1). There are currently over 800 different definitions for the term [21]. In this paper, we focus on areas larger than 5 ha with trees higher than 5 m and minimum 50% canopy cover (on a reference area of 30 × 30 m). Such a type of tree cover will typically provide diverse niche spaces and a multitude of ecosystem services (natural forest), and/or a high density of resource (plantation). These criteria are more conservative than those employed in many other definitions, but we believe that it is important to understand what is happening to this type of resource. Note that we use the term ‘tree cover’ instead of ‘forest’ to indicate that our analysis does not focus on natural forests but encompasses all types of tree cover, including plantations.

## 2. Material and methods

### (a) Overview

To estimate historical tree cover loss (from approx. 8000 BCE (Before Common Era)–2000), we developed a global habitat suitability model for tree cover, and compared the results with tree cover observed in 2000 [17,18]. To evaluate recent tree cover change (2000–2012) globally and in China, we used the Hansen *et al.* [17] data. These detect changes from minimum 50% crown cover to approximately 0% crown cover (= loss) and from approximately 0% crown cover to minimum 50% crown cover (= gain). As these data do not capture the gradual processes of expansion and maturation of existing stands and degradation (e.g. selective logging), we also analysed Landsat Tree Cover Continuous Fields data [18] for China, which quantify crown cover for vegetation higher than 5 m on a continuous scale in both 2000 and 2010. Finally, we reviewed the extent of forest cover (gains) using eight additional datasets (electronic supplementary material, table S2), which employ different forest definitions.

### (b) Data

The Hansen *et al.* [17] and Sexton *et al.* [18] data used to evaluate recent tree cover changes (2000–2012) are high-resolution maps (30 m) derived from MODIS and Landsat data. In these, ‘tree cover’ is defined as all vegetation taller than 5 m in height (including tree crops and plantations). More specifically, the detection algorithm records canopy cover as per cent light intercepted by the canopy (and not simply crown extent; M. C. Hansen 2016, personal communication). The Sexton *et al.* [18] data record crown cover on a continuous scale for several time slices, and here, we focus on a minimum canopy cover threshold of 50% (for more details, see electronic supplementary material, Methods S1). Whereas the Hansen *et al.* [17] data record percentage crown cover for 2000, for subsequent years, these data only record the presence of ‘losses’ and/or ‘gains’, whereby loss is defined as ‘a stand-replacement disturbance or the complete removal of tree cover canopy at the Landsat pixel scale’ (i.e. from minimum 50% to approximately 0% canopy cover), and gain as ‘the establishment of tree canopy from a non-forest state’ (i.e. from approximately 0% to minimum 50% crown cover). In our calculations, we excluded all pixels that experienced both tree cover losses *and* gains to minimize the signal from regular forestry practices in the analysis.

For all other data used in this paper, see the electronic supplementary material, Methods S2 and table S1.

### (c) Data validation

We validated the accuracy of the tree cover datasets for China using a stratified random sample of 1685 [17] and 862 [18] cells of 100 × 100 m with very high-resolution imagery and photos in Google Earth. The overall accuracy for both datasets was greater than 87%. Omission errors marginally exceeded commission errors, but the majority of areas where tree cover was omitted contained scarce, scattered or low-height tree cover. Consequently, while the data are less sensitive to detecting change in small-scale patches of tree cover and/or areas with scarce tree cover, we consider them relatively robust for large (greater than 5 ha) contiguous blocs of trees with crown cover of minimum 50% (the focus of this paper). For more details, see the electronic supplementary material, Methods S3 and Results S1.

### (d) Statistics

To compare potential historical tree cover with present-day tree cover, we modelled habitat suitability for tree cover (crown cover of 50% or more) using a species distribution modelling approach. The input consisted of 10 000 random spatially balanced points of minimum 50% crown cover in 2000 [17] and 26 candidate predictor variables representative of climate, topography and soil type. The modelling procedure was repeated 10 times, each time using another random sample of input points, to ensure that the results were not influenced by the selection of records. For details on model development, selection and validation, see the electronic supplementary material, Methods S4.

To characterize the two-dimensional niche space of investment, afforestation and remotely sensed tree cover gains, we undertook a principal components analysis (centred and scaled to unit variance) of all numerical (*N* = 25) environmental variables. We then mapped the amounts of investments and afforestation and tree cover gains into that space. As no finer-scale data were available, we assigned the total investment and afforestation per forest programme in equal proportions to the target provinces. The analysis thus does not capture that some target provinces may have received more funding than others, and that within provinces funding and afforestation may have focused on particular environments. All figures given in this paper are based on the most high-resolution data available: for recent tree cover changes 30 m resolution, and for historical losses 1 km resolution.

## 3. Results

### (a) Historical losses of dense tree cover (crown cover of minimum 50%)

**Figure 2.**
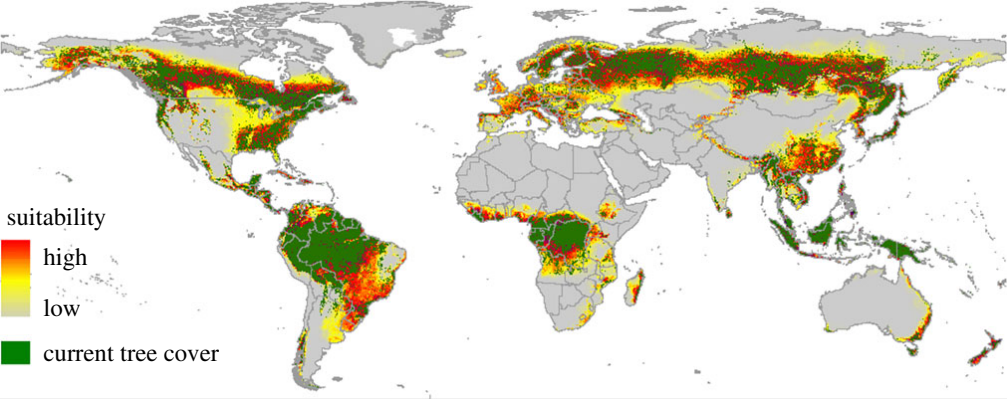
Potential historical loss of tree cover with minimum 50% canopy cover. Predicted global distribution of macro-environments that are suitable for tree cover with minimum 50% canopy cover (yellow to red) and actual tree cover with minimum 50% canopy cover in 2000 (green).

In total, 57–67 million km^2^ of the planet's terrestrial surface is suitable for dense tree cover (higher than 5 m and minimum 50% crown cover of 50% or more), and we estimate that total historical losses may have amounted to 24–34 million km^2^ (42–51%). The big planetary losses are located in the US Midwest, Brazilian dry and Atlantic forests, W Europe, Turkey, Russia, W Africa, temperate E China, India and E Australia (figure 2), which are all areas that are characterized by agricultural intensification.

**Figure 3.**
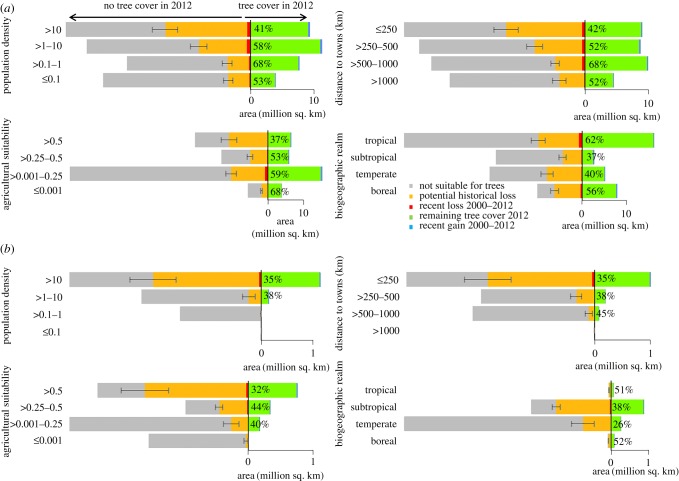
Tree cover changes globally (*a*) and in China (*b*). The analysis is restricted to tree cover with minimum 50% crown cover. The percentages show total remaining tree cover in 2012 (mean modelled remaining tree cover + recent gains), and the error bars indicate the range of predictions of the tree cover suitability models (see electronic supplementary material, Methods S4). For detailed figures of recent tree cover losses, see the electronic supplementary material, figures S4 and S9, and for associated maps see electronic supplementary material, figure S10.

Biogeographically, temperate and subtropical forests have been most affected, with estimated historical losses of approximately 50–60%. Tropical areas have been affected least (figure 3*a*).

**Figure 4.**
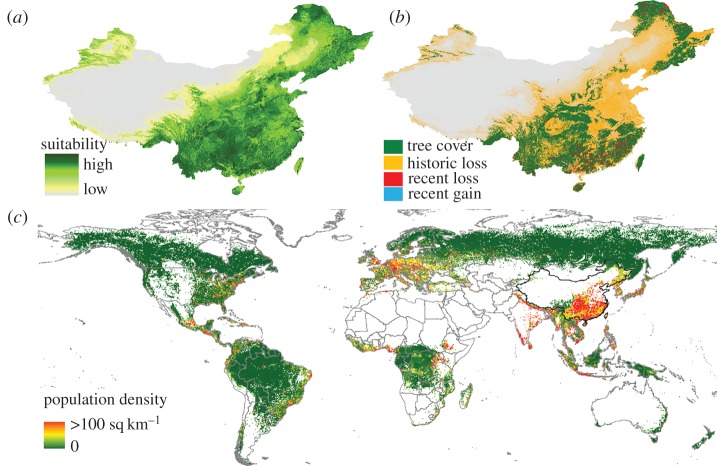
Tree cover (with a minimum of 50% crown cover) losses and population pressure. (*a*) Predicted historical tree cover distribution in China (approx. 8000 BCE; based on climatic suitability). (*b*) Tree cover in 2000 and recent losses and gains. (*c*) Global tree cover in 2000 shaded by population density.

China has approximately 3.2–4.1 million km^2^ environmental space suitable for tree cover with minimum crown cover of 50%. Our model suggested that historical losses amount to 59–67% (approx. 1.9–2.7 million km^2^) (figure 4). These figures underestimate actual losses of natural forests as approximately one-third of China's tree cover today consists of plantations [22]. The greatest absolute loss occurred in the subtropics (greater than 1 million km^2^). Relative losses were largest in temperate E China (Yellow River Basin)—the cradle of the Chinese civilization where most of China's agriculture, population and urban expansion are concentrated. Today, only 26% of China's temperate tree cover remains (figure 3*b*).

### (b) Recent changes in dense tree cover (crown cover of minimum 50%)

Globally, recent losses of tree cover of minimum 50% crown cover amount to another 5% (approx. 1.7 million km^2^), with a *net* loss (loss-gains) of approximately 3% (approx. 1 million km^2^). Recent losses have been highest in the tropics and recent gains have been highest in the boreal regions (figure 3*a*) [17].

In China, recent losses totalled approximately 50 000 km^2^ (4%), with a net loss of approximately 33 000 km^2^ (2.5%). Thus, proportionate total and net losses in China were only marginally below the global average. However, these data do not capture the gradual processes of regrowth of longer-lived existing stands and degradation (e.g. selective logging). Using the Sexton *et al.* [18] data, we calculate that a total area of 252 900 km^2^ experienced a drop in crown cover from a minimum of 50% to less than 50% between 2000 and 2010, while 285 869 km^2^ had an increase from less than 50% to minimum 50% crown cover (electronic supplementary material, figure S1). Thus, overall, there has been a small net increase (approx. 33 000 km^2^) in the area with minimum 50% crown cover. However, the vast majority of these incremental gains were small, with over one-third of the pixels that crossed the threshold of 50% crown cover having experienced an increase by less than 10%. Around two-thirds experienced an increase by less than 20%, and only 5% by 50% or more (electronic supplementary material, figure S2).

Combining historical and recent losses, we estimated that China lost 60–68% of its tree cover—more than any other major forested country (with a forest area 1 million km^2^ or more). China may once have had the fifth largest forest area in the world, but its forest area has now been superseded by that of the Democratic Republic of the Congo and Indonesia, which have ‘only’ lost an estimated 27–29% and 17–18% of their original forest area, respectively.

### (c) Recent changes in sparser tree cover (less than 50% crown cover)

In order to understand what type of tree cover China may be gaining, we reviewed tree cover estimates provided by eight additional datasets (electronic supplementary material, figure S3). The largest gains are those reported by China to the FAO (more than 43 000 km^2^ yr^−1^). The FAO uses a land-use-based definition for the term ‘forest’, which includes immature plantations and bare land if it is to be afforested. At maturity, the vegetation must reach more than 10% crown cover, higher than 5 m, bigger than 0.5 ha area and more than 20 m width. In its National Forest Inventories, China applies a definition based on actual land cover with a crown cover threshold of 20% minimum, and includes special purpose shrubs. According to these data, tree cover has been increasing by c. 33 000 km^2^ yr^−1^. With a crown cover threshold of 50% minimum, the detectable net gains in the data generated by Sexton *et al*. [18] were approximately 3300 km^2^ yr^−1^. The data generated by Hansen *et al.* [17], which only register substantial changes (from a minimum of 50% to approximately 0%, and vice versa), record a net loss of tree cover of approximately 2800 km^2^ yr^−1^. The discrepancies in the reported gains are thus to a large extent consistent with the respectively applied forest definitions. However, it is noteworthy that the overall detectable net gains of cells with a minimum of 50% canopy cover and height greater than 5 m, hence gains of dense tree cover, according to both Sexton *et al.* [18] and Hansen *et al.* [17], are more than an order of magnitude less than the gains reported in China's National Forest Inventories. For further details and other datasets, see electronic supplementary material, Results S2.

### (d) Detectable tree cover gains versus afforestation effort

**Figure 5.**
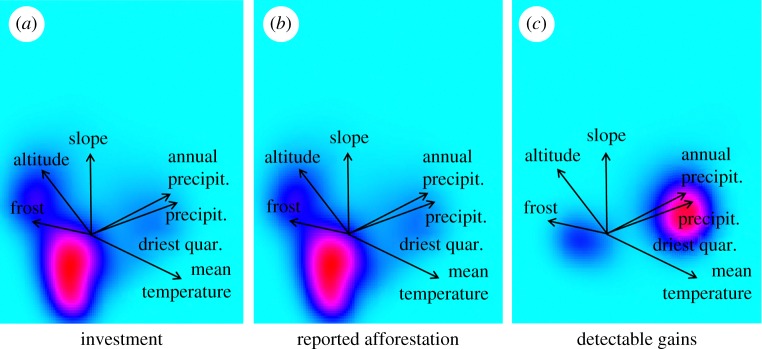
Province-level analysis of investment, reported afforestation and observed tree cover gains (2000–2012) in climatic space. The two-dimensional representation of China's climate space was generated using a PCA. Red colours indicate high investment, respectively afforestation and gains in that particular part of the climate space. Panels (*a*) and (*b*) are province-level averages and therewith indicative only (see §2).

According to the statistical yearbooks, China planted a total of 1.17 million km^2^ with trees between 1992 and 2013. The yearly average was 55 000 km^2^, and in any given year, at least 25 000 km^2^ have been planted (see electronic supplementary material, Results S3). To look more closely at whether high investments coincide with substantial detectable gains (measured using Hansen *et al.* [17]), we conducted a province-level analysis and mapped (i) afforestation investment, (ii) reported amounts of afforestation, and (iii) detectable tree cover gains into a two-dimensional representation of China's environmental space (figure 5). Between 2000 and 2010, China invested the most money and also reported the largest afforestation in provinces that are marginal and characterized by aridity, low temperatures, high altitudes and steep slopes (e.g. in China's West at the upper reaches of the Yellow and Yangtze Rivers). However, the vast majority of the substantial tree cover gains (figure 5*c*) have occurred in areas with more rainfall and less extreme low temperatures, but considerably less financial investment (e.g. in China's SE and NE). In total, 97% of all these gains have occurred in areas that our model classed as suitable for tree cover and that—at a province level—may have received less than 50% of the investment. In the less suitable main target areas of Chinese afforestation programmes, detectable gains in terms of dense tree cover were limited (which does not preclude successful afforestation with shrubs or narrow strips of trees or slowly maturing stands; see §4).

### (e) Socio-economic correlates of tree cover change

At a global scale, both historical and recent tree cover loss followed clear spatial and macro-economic patterns. Spatially, losses have disproportionately affected areas with high population pressure and easy access (figure 3*a*; electronic supplementary material, figure S4). Consequently, today most (71%) tree cover remains within areas with lower human population pressure. In low-income countries (with a GDP *per capita* of less than US$ 10 000), a large fraction of tree cover still coincides with areas of high population pressure, where it is currently being lost at an alarming rate: between 2000 and 2012 approximately 25 000 km^2^ yr^−1^ (6%) (electronic supplementary material, figure S5). A slightly different picture emerges for high-income countries with a large proportion of boreal forests (electronic supplementary material, figure S6): here, there have also been substantial tree cover losses at distances of more than 1000 km from towns and in areas of very low population density, reflecting the proneness of boreal forests to natural loss dynamics (e.g. caused by fire, storm, insects and pathogenic fungi). Protected areas appeared to hold tree cover losses, but only to an extent: recent proportionate tree cover losses outside protected areas were approximately twice as high as inside (1.6 million km^2^ (5%) versus 94 000 km^2^ (2.9%); electronic supplementary material, figure S6).

**Figure 6.**
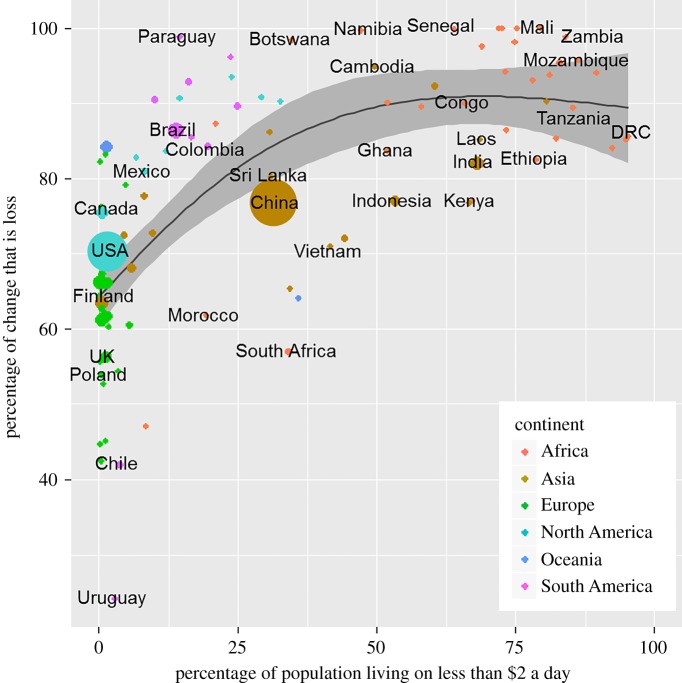
Proportionate net tree cover loss by country against poverty. The size of the plotting symbols is proportionate to the absolute increase in GDP between 2000 and 2012. The trend line is based on natural cubic splines (*F* = 42; d.f. = 97; *R^2^* = 0.5; *p* ≤ 0.001) and the grey shading shows the 95% confidence intervals. The analysis is restricted to countries with a minimum of 10 000 km^2^ tree cover in 2000. Exemplar countries are listed. Proportionate net tree cover loss = loss/loss + gain.

The proportion of tree cover losses not compensated by within-country tree cover gains (loss/loss + gain) was highest in countries with high levels of poverty, urbanization, population growth, a high GDP reliance on agriculture and with expanding food production (figure 6; electronic supplementary material, figures S7 and S8). There was a strong continental signal whereby the fraction of uncompensated losses was particularly high in African countries. Losses appeared to be lower in SE Asia, but this hides the fact that there large amounts of natural tree cover are replaced with tree cash crops such as rapidly growing oil palm, rubber and *Eucalyptus*. There was a small negative correlation (*R* = −0.21, *p* ≤ 0.001, d.f. = 176) between the proportionate historical tree cover loss (per country) and the recent proportionate uncompensated loss, indicating that countries that have historically deforested their territories may now have shifted to protecting the remaining tree cover.

The global picture of tree cover losses illustrates China's challenging situation: China has one of the world's greatest coincidences of tree cover and people (figure 4), with 88% of China's tree cover located in areas with more than 10 people per km^2^ and 33% in areas with greater than 100 people per km^2^. Globally, only 29% and 6% of tree cover are located in these population density classes, respectively. Our calculations showed that China also has one of the world's highest correlations between area suitability for tree cover and for agriculture (globally *R* = 0.35; *p* ≤ 0.001, d.f. = 8 318 389; in China *R* = 0.73, *p* ≤ 0.001, d.f. = 546 123; China ranked ninth in the world in terms of this correlation), and the zone that is suitable for tree cover therefore overlaps to 84% with the zone suitable for agriculture. Finally, China has one of the world's lowest per person areas of suitable agricultural land (approx. 3 × 10^−3^ km^2^ per person, which is less than one-third of the global average). As is the case globally, both historical and recent tree cover losses in China have mostly affected areas with high population pressures, high agricultural suitability and easy access (figure 3*b*). Today, most of China's tree cover remains on sloping lands (1–10°), while in flat areas as much as approximately 90% of original tree cover may have been lost (electronic supplementary material, figure S9). Between 2000 and 2010, approximately 10 000 km^2^ of tree cover have been lost from large and unbroken expanses of intact natural forest areas [23] (see electronic supplementary material, Results S4), and between 2000 and 2012, almost 3000 km^2^ of tree cover with crown cover of 50% or more has been lost from protected areas (3.2%). This was slightly higher than the global proportionate loss from protected areas (2.9%) and similar to the proportionate loss outside protected areas in China (4%), raising concerns over protected area efficacy.

## 4. Discussion

A realistic understanding of China's forestry situation is important as China is responsible for a large part of the world's ‘real estate’, and imports more timber and exports more wood products than any other country [24]. Our analyses provide a global picture of tree cover loss, which we use to contextualize tree cover change in China. Globally, our model-based estimate of 42–51% historical net tree cover loss (minimum of 50% canopy cover) is concordant with previous estimates compiled from a mixture of regional and global biome maps [25]. Added to this is another 5% (relative to 2000) of global tree cover loss between 2000 and 2012 detected from remotely sensed data (with a net loss of 3%) [17]. In accordance with forest transition theory [26] and general expectations, our analyses show that countries characterized by rapid expansion, high population pressure, poverty and an emphasis on food production exert particularly unsustainable pressure on their tree cover. Both historical and recent losses have been highest in densely populated areas and on agriculturally suitable land. Consequently, today most tree cover remains in areas with less high pressures. This gradient—high losses in areas of high population density and lower losses at farther distance—is particularly steep in the tropics and low-income countries, which have experienced the least historical losses and are now in the line of fire. The tree cover gains largely mirror this pattern. In low-income countries, recent gains were largest in areas of high population density and agriculturally suitable land, whereas in high-income countries, recent gains were largest in areas at moderate distances to towns and on less agriculturally suitable land (e.g. in boreal regions).

The global context shows that China is highly predisposed to tree cover losses. Previous reports have indicated a positive response to this challenge, and conclude that China has reached a stable forest transition [5,6]. Chinese forest plantations have a reported increase in area that is higher than the rest of the world combined—with a net increase of almost 20 000 km^2^ yr^−1^ between 1990 and 2000, and almost 30 000 km^2^ yr^−1^ between 2000 and 2010 [7]. Yet, the high-resolution remotely sensed data analysed here, which measure vegetation higher than 5 m and with a minimum of 50% crown cover, show only modest net gains.

Our work highlights that China's forest cover gains are highly definition-dependent. If ‘forest’ is defined according to the FAO criteria (including immature and unstocked areas), China's forest cover gains between 2000 and 2010 were larger than the combined area of Germany, The Netherlands, Belgium and Luxembourg [7]. If forest is defined according to China's own criteria (see below), China has gained an area smaller than size of Germany; and if forest is defined according to what non-specialists would view as forest (contiguous blocs of tall (higher than 5 m) and closed (minimum 50%) crown cover), the detectable gains are smaller than the size of The Netherlands.

Today, China's definition of forest uses a 20% crown cover threshold (modified from 30% in 1994), and there is no explicit height threshold. Many of China's plantations are young and may not (yet) have reached more than 5 m height. Likewise, plantations aiming to stabilize eroding soils do not necessarily need to reach higher than 5 m to be successful. Indeed, in 2004, China expanded its definition of forest to include ‘special purpose scrubs’ that are planted in dry areas or at high elevations for environmental conservation [27], and much of the reported successes are shrub plantings [28]. In addition to tree height and crown cover, the size of the area and that of the reference area for crown cover are also important [29]. We noted that the accuracy with which patches of tree cover were detected increased substantially with the size of the area. Detection is good for areas larger than 5 ha, whereas the FAO and many other definitions use a lower area threshold of 0.5 ha. In summary, owing to different definitions, there is not necessarily a conflict between the large gains reported by China to the FAO or in its National Forest Inventories and the modest gains reported here.

In addition to definitions, there are technical issues that need to be kept in mind. Even if the tree cover is higher than 5 m, if the tree cover is scarce and/or consists of small-scale patches, it is difficult to detect with remote sensing. For instance, narrow forest belts planted in the desert as promoted by the ‘Three-North’ Shelterbelt Program are difficult to pick up from satellite imagery (M. C. Hansen 2016, personal communication). Our validation analyses of the Hansen *et al.* [17] and Sexton *et al.* [18] data showed that the tree cover omission error rate marginally exceeded the commission error rate (see §2 and electronic supplementary material, Results S1), and there were some larger omissions in challenging environments (e.g. arid environments, areas with snow cover and/or steep slopes)—all areas where China is planting trees. However, the majority of omission affected patches of tree cover smaller than 5 ha.

Thus, even taking a conservative view of the data and recognizing that an enormous amount of afforestation effort may be around shrubs or narrow strips of trees, it is noteworthy that the data show that *at best* there have only been moderate gains of large contiguous blocs (larger than 5 ha) of vegetation higher than 5 m and with a minimum of 50% canopy cover, i.e. tree-dominated habitats as a source for timber and other forest-related ecosystem services. Closed forest cover, as defined in this paper, is still being lost at a high rate—including from protected areas. In addition, the data analysed here do not distinguish between natural forests and plantations. Rubber, pulp, fruit tree and *Eucalyptus* plantations have replaced large amounts of natural forest in China [19]; thus, the losses of natural forest will have been significantly larger than reported here.

Given China's population size and rate of economic growth, it is perhaps surprising that the situation is not worse than quantified here. There is an increasingly acute conflict between the land needs for massive economic growth, food, timber and conservation. China's demand for wood is continuously growing and expected to reach 457–477 million m^3^ in 2020 [12]. Likewise, China has to feed one-fifth of the global population on less than one-tenth of the globally usable agriculturally suitable land, and there is an increasing water scarcity problem [30]. Consequently, China's agricultural policies focus on large-scale intensification, which results in losses of tree cover from rural lands to facilitate the use of large-scale machinery.

To spare prime land for agriculture, and to ameliorate environmental degradation, afforestation efforts have often focused on marginal areas—e.g. arid and/or sloping land in W China. However, the detectable fast and substantial tree cover gains have mainly occurred in E China on agriculturally suitable land. Thus, returns for the large-scale tree planting investment in marginal areas may be low, or take a long time to materialize. Stunted growth (trees reaching only approx. 20% of their natural height after 30 years of planting) have been observed in many marginal areas [10]. Problems may have also arisen due to the choice of planting material—often single species or single clones that are fast-growing and possibly poorly adapted to local conditions [3]. Even if the aim of planting is not to have trees higher than 5 m numerous reports on planting failures give cause for concern [10,15,31]. Several studies [32–34] found large differences between reported and actual areas of afforestation in the marginal areas targeted by the ‘Three-North’ Shelterbelts and Grain for Green programmes, and the estimated overall survival rate of trees in afforestation projects from 1952 to 2005 reportedly was approximately 24% [3]. The tendency for monoculture planting increases susceptibility to disease [35]—for instance, a poplar beetle outbreak in the 1980s in Ningxia affected 90% of the trees and approximately 80 million trees—planting efforts of two decades—had to be cut down [36]. Even where tree planting is successful in marginal areas, several studies have also cautioned that the large-scale afforestation in areas that are not naturally suitable may not ameliorate but instead exacerbate problems such as water shortage, erosion, and dust and sand storms due to the comparatively high evaporative demands, deep rooting systems and shading (exclusions of grasses), which lead to desiccation, top-soil vulnerability and vegetation mortality [3,10,37,38].

More positively, the six key forest programmes have achieved some major successes such as greatly reduced logging in the target provinces of the National Forest Protection Program, and a massively expanded protected forest area network [11]. In the recent past, China has placed an increasing emphasis on natural forest protection, increasing the plantation area in suitable regions and afforesting with indigenous and/or ecologically suitable species [39]. Consequently, tree survival rates may already be improving [40] and plantations may now start to provide returns. China has over 5000 years of history of human impact on the environment, and viewed over millennia China is *en route* to forest recovery.

## 5. Conclusion

China's investments into halting and reversing tree cover loss clearly have ameliorating effects and without these, the situation would likely deteriorate rapidly. It is unequivocal that China's biomass and consequently, its carbon sinks are increasing [41,42]. However, planting trees is not the same as gaining forests. The current best available evidence, albeit with some uncertainty, suggests that China's tree cover gains may to a large extent consist of low-height, sparse and/or scattered plantations. There are fewer gains of contiguous blocs of high and closed tree cover. Our results support the need for more refined monitoring and reporting of forest areas globally [19,29], and systematic nation-wide monitoring of plantation performance in relation to climatic suitability in China. The massive investment into afforestation in environmentally marginal areas may not be leading to rapid returns and it will also likely require continued input. That input in marginal areas could be balanced against integrated land-use planning for trees and food in the more environmentally suitable areas. China is already taking a range of steps to maximize returns for the current investment. Our results highlight the benefits of continued implementation and increased adoption of these strategies; including: (i) protection of the few remaining natural forests, (ii) carefully designed afforestation strategies that focus on suitable areas, ecologically appropriate and/or native species, (iii) timber certification schemes for both imports and domestic production, (iv) interconnected instead of separated agricultural and forestry strategies and integrated land-use for food production and trees, and (v) if there is a need for continued tree planting into suboptimal and marginal areas—recognition of the potential for very delayed returns on investment.

## Supplementary Material

Supplementary material

## Data accessibility

The datasets supporting this article are available at http://www.rbge.org.uk/science/genetics-and-conservation/antje-ahrends-homepage.
