# Supplementary material for: China's fight to halt tree cover loss
Source: Proc Biol Sci. 2017 May 3;284(1854):20162559. doi: 10.1098/rspb.2016.2559 (PMC5443932; doi:10.1098/rspb.2016.2559)
Supplement: Supplementary material [file rspb20162559supp1.doc]

**Supplementary material - China’s flight to halt tree cover loss**

*A Ahrends, PM Hollingsworth, P Beckschäfer, H Chen, RJ Zomer, L Zhang, M Wang, J Xu*

**Supplementary Note 1: China’s key forestry programs**

China’s six key forestry programs cover 97% of China’s counties and have a combined afforestation aim of 760,000 km^2^. Their scale is globally and historically unparalleled [1].

**The Key Shelterbelt Development Programs** (1978 – 2050) comprise several sub-programs including the Three North Shelterbelt Program (TNSP), the Shelterbelt Development along the Middle and Upper Reaches of the Yangtze River, the Coastal Shelterbelt Development Program, and the Farmland Shelterbelt Network in the Plains Areas. The TNSP covers 42% of the Chinese territory across 13 provinces in China’s NW, N and NE regions. Its main aim is to reduce and control desertification and erosion in N China by planting shelterbelt forests around farmlands and pastures, and afforesting barren land. Between 2001 and 2010 275,000 km^2^ have been afforested [2] and tree coverage in the region has reportedly increased from 5% to >10% [3]. This has included a large citizen participation component with citizens planting a total of 66 billion trees since 1978. The total planned afforestation by 2050 is 356,000 km^2^. Investments between 2002 and 2006 have totalled RMB 60 billion. The shelterbelts are between ~250-550 m wide, with a fence along the perimeter, and the vegetation is planted in a chessboard pattern.

**The Grain for Green Program** (2000 – 2020 with previous pilot phase) aims to convert croplands and barren land to forests in environmentally fragile areas (e.g. steep slopes) to reduce erosion, control flooding and provide forest resources in 25 provinces in central and western China. The converted lands are generally located in environmentally marginal areas (e.g. on sandy soils) where yields are low. The program resulted in a total afforestation of 248,600 km^2^ between 1999 and 2012 and targeted 32 million households [4]. Investments (grain and cash subsidies paid to farmers) totalled RMB 180.54 billion between 2000 and 2009 [3]. In 2007 a decision was made to increase the total investment into the program to RMB 430 billion.

**The Natural Forest Protection Program** (2000 – 2020) aims to protect and rehabilitate natural forests and to increase forest resources. The program covers 17 provinces along the Upper reaches of the Yangtze and Yellow Rivers, in China’s NE and in Inner Mongolia. Between 2001 and 2010 44,000 km^2^ of new forest was established [2]; 120,000 km^2^ of land was set aside for natural regeneration; >1 Mio km^2^ of forest was taken into management and protection; and >600,000 displaced forester workers have been re-settled [3]. Commercial logging in natural forests has been banned within key state forest areas, and timber harvesting has consequently been reduced from 18.24 Mio m^3^ in 1997 to 10.99 Mio m^3^ in NE China and Inner Mongolia [5]. Total investments between 2000 and 2010 were RMB 118.6 billion [6], and another RMB 244 billion has been allocated for the 2^nd^ program phase between 2010 and 2020 [5].

**The Sandification Control Program in Beijing and Tianjin Vicinity** (2001 - 2023) aims to reduce desertification and dust storms in Beijing and surrounding areas through various means, including 52,000 km^2^ of afforestation and 20,000 km^2^ of grass establishment between 2001 and 2010^2^, with a total investment of RMB 21.84 billion [3]. The program covers five provinces.

**The Wildlife Protection and Nature Reserve Development Program** (2001 – 2050) implemented in 32 provinces aims to conserve endangered species and habitats through the expansion of China’s nature reserves network to a coverage of 1.55 Mio km^2^, or ~16% of China’s total land area. Investments totalled RMB 1.57 billion between 2001 and 2004 [1].

**The Fast-Growing and High-Yielding Timber Base Construction Program in Key Areas** (2001 – 2015) aims to reduce the timber supply shortage. It covers 18 provinces in the east of China (with total annual rainfall of ≥400 mm). The target is to plant 130,000 km^2^ of fast-growing and high-yielding timber to provide 133 Mio m^3^ timber annually – equivalent to 36% of China’s commercial timber consumption and almost sufficient to balance the current demand-supply gap of c. 150 Mio m^3^ [5]. Until 2007 49,000 km^2^ had already been established. While all other key forestry programs are government led and mainly government financed, major financing for this program comes from the commercial sector. Total investments in 2004 were RMB 205.6 million [1].

**Supplementary Methods 1: Landsat Vegetation Continuous Fields data**

We generated maps depicting vegetation cover ≥50% from Landsat Vegetation Continuous Field (LVCF) data (Fig. S1). LVCF is a global dataset providing estimates of the percentage covered by vegetation >5 m in height per 30 × 30 m pixel. It is derived by means of a regression tree algorithm that rescales Moderate-resolution Imaging Spectroradiometer (MODIS) Vegetation Continuous Field data [7] from 250 to 30 m resolution based on the Global Land Survey (GLS) Landsat datasets [8]. LVCF data are available for the nominal epochs 2000, 2005 and 2010 and can be downloaded for free from the Global Land Cover Facility server (http://glcf.umd.edu/data/landsatTreecover/). For mapping we applied a threshold of 50% to the 2000 and 2010 LVCF data using the statistical software R [9]. Subsequently LVCF tiles were re-projected from UTM to Albers equal-area conic projection, mosaicked and cropped to the boundary of China with ArcMap software by ESRI.

We note that the areas with ≥50% crown cover provided by Sexton *et al.* [10] are on the lower side compared to other datasets. It is possible that the coarser resolution of the underlying MODIS data may lead to an underestimation of dense tree cover. Consequently, caution has to be applied when looking at absolute areas for dense tree cover, particularly where blocs of tree cover are smaller than the spatial resolution of the MODIS data (250 × 250 m). However, the changes in tree cover (losses and gains) in larger blocs of tree cover – the focus of this paper – can be inferred (see Supplementary Results 1). We also conducted an analysis of acquisition dates of Landsat images used in the computation of LVCF data (Fig. S2; dates were obtained from http://earthexplorer.usgs.gov/). In total, 89% of the images in 2000 und 90% of the images in 2010 were acquired during the growing season. For the vast majority of China the 2010 images were acquired in Jun-Aug whereas in 2000 a number of images were acquired in May and Sep (i.e. at the beginning and end of the growing season). Thus, if anything, gains between 2000 and 2010 could be overestimated (but we record only modest gains). One exception to this are the seasonally dry tropical areas (Xishuangbanna) for which there were more images from the dry season (Jan-Feb) in 2010 than in 2000. In this area gains of deciduous trees (e.g. *Hevea brasiliensis* – rubber), which shed their leaves during the dry season, may be underestimated.

Note that the MODIS VCF data tend to be less reliable for crown cover thresholds ≤20%, and that both the MODIS VCF and the LVCF data tend to be compressed in the intermediate range with fewer extreme values (0% and 100%) [10]. We consequently do not use these data to analyse changes for lower crown cover thresholds; nor do we evaluate their accuracy for detecting a lack of tree cover.

**Supplementary Methods 2: Environmental and other data**

The environmental data used in this paper comprised 26 bioclimatic variables representative of climate, topography, and soil (Table S1). Altitude was derived from the Shuttle Radar Topography Mission SRTM digital elevation model [11] at a resolution of ~90 m (3 arc seconds). Slope was calculated from the altitude layer using the library ‘raster’ [12] in R 3.1.0 [9]. Climate data were derived from several datasets: we used global interpolated climate surfaces based of weather station data from 1950-2000 [13] at a resolution of ~1 km (30 arc seconds) for temperature and precipitation related variables. From this we also calculated the number of very dry months with <3 mm rainfall. Potential evapotranspiration, actual evapotranspiration, soil water content, and water stress (precipitation over potential evapotranspiration) were obtained at a ~1 km resolution [14, 15]. Monthly mean sunshine hours (as percent of possible maximum sunshine), ground frost frequency, and monthly mean wind speed at 10 m above ground were obtained at a resolution of ~19 km (10 arc minutes) [16]. Finally, the dominant soil type was obtained from the Harmonized World Soil Database Version 1.21 [17]. All layers were resampled to ~19 km and ~1 km resolutions (based on the mean and bilinear interpolation for continuous variables, and a nearest neighbour function for categorical variables). All raster processing, equal area corrections and calculations were done using the library ‘raster’ [12] in R 3.1.0 [9]. Uncertainty in all of these layers should be noted, particularly in soil type, wind speed, and precipitation related variables, and also in the CRU CL 2.0 layers [16] because these were interpolated to a higher resolution. Generally, uncertainty in all these layers is highest in mountainous areas, and areas where there are few weather stations [13].

Other GIS layer used in this study included a layer on urban areas [18] to calculate distance to, and growth rate (2000 – 2010) of, the nearest town. We further obtained layers for protected areas of IUCN category I-V [19], population density [20, 21], biogeographic realms [22] and agricultural suitability [23]. All layers were converted to raster format and resampled to resolutions of ~1 km and ~5 km for subsequent analyses.

**Supplementary Methods 3: Accuracy assessment for recent tree cover change maps**

We validated the accuracy of the Hansen *et al.* [24] maps of recent tree cover losses and gains for China using a stratified random sample of 1,485 cells of 100 × 100 m. The validation sample cells had a coarser resolution than the actual data (30 × 30 m) to account for potential image registration errors. The following sample sizes were assigned to the individual strata: gain (400 cells), loss (400 cells), and no change (685 cells). Of the latter, 217 cells had been classed as having no tree cover, and the remaining cells represented with approximately equal proportions tree cover in four quantiles: <25%, 25-<50%, 50-<75%, and 75-100%. We used a subsample of the same points plus an additional 133 points to validate the accuracy of the Sexton *et al.* [10] data for tree cover in 2010 and gains and losses between 2000 and 2010. The samples were validated using very high resolution imagery in Google Earth and, where available, photos shared via Panoramio. Specifically, we noted the presence and absence of tree cover in the reference cells, and for presences visually estimated canopy cover per cell in two categories: 1-<50% and ≥50%. The photos, where available, provided auxiliary information during image interpretation. We used standard statistical methods [25] to calculate error matrices and accuracy statistics.

In order to test whether the maps by Hansen *et al.* [24] may be prone to omitting tree cover and tree cover gains we conducted an additional targeted assessment of 200 cells of ‘contentious’ areas. These are areas where other recently published high-resolution landcover maps report forest cover and/or gains and the Hansen *et al.* data do not. The other dataset included:

- China National Landcover Map for the year 2005 (Landsat based; 100 m resolution) [26]
- ALOS PALSAR-2/PALSAR forest/non-forest map for the years 2007 and 2010 produced by the Japanese Aerospace Exploration Agency (Jaxa) (25 m and 100 m resolution) [27]
- GlobeLand30. Landsat based global landcover map for the year 2010 produced by the National Geomatics Center of China (30 m resolution) [28]. We validated the accuracy of this dataset in China using another random sample of 500 cells.

Wherever possible the reference data (Google Earth and photos) were gathered for the relevant two time periods to validate change, e.g. in the case of the data produced by Hansen *et al.* for 2000 and 2012. Such data points, and data points for which imagery was available for 2000-2004 and 2010-2012 received a weight of 1. These time windows were chosen because there was often no high resolution imagery for the precise assessment years (particularly for 2000). We applied appropriate caution when interpreting the images – e.g. if there was evidence for recent changes (fresh clearing, young plantation) in images for 2001-2004 these were not regarded as representative for the baseline in 2000, and not included in the validation. Data points were also included but received a lower weight when the following circumstances applied: (1) when reference imagery was available for 2000-2004 and only for post 2012, but when the post 2012 land cover was unlikely to have changed since 2012 (weight = 0.75); and (2) when imagery was only available for 2010 or later but where there was clear evidence for a given landcover/change (weight = 0.5) – e.g. a fresh clearing (loss), a recently established plantation (gain), or a mature forest with no evidence of disturbances at the point or in surroundings areas (no change). In total, 35% of the data points had complete data for both time periods. We calculated the error matrices and accuracy statistics both with and without applying weights.

**Supplementary Methods 4: Modelling suitable environmental space for global tree cover**

We randomly selected 10,000 spatially balanced cells that according to the data generated by Hansen *et al.* [24] had crown cover ≥50% in 2000. These records were combined with environmental surfaces (Table S1) to produce spatially explicit models of habitat suitability for tree cover using a Species Distribution Modelling approach [29]. The procedure was repeated 10 times, each time using another random sample, to ensure that the results are not influenced by the selection of records. We used the suite of models implemented in the R library ‘dismo’ [30], of which MaxEnt [31] performed best. Model performance was measured in terms of accuracy of predictions and response curves (expert opinion), and the Area Under Curve (AUC) of the Receiver Operating Characteristic (ROC) for test data under 10-fold cross validation. We used two thresholds for converting the continuous probabilities to binary data: (1) a threshold that mainly focusses on minimizing the rate of false negatives (specifically, that minimizes 6 * training omission rate + 0.04 * cumulative threshold + 1.6 * fractional predicted area), and (2) a threshold that results in equal entropy of thresholded and original distributions. Thresholds that maximize sensitivity plus specificity tended to under-predict the area of suitable habitat, and were therefore discarded. We calculated the mean and the range of the predictions produced with the two selected thresholds.

MaxEnt has an inbuilt regularization method (L1-regularization) for predictor selection and performs reliably in the presence of correlated variables [32]. However, there is a risk that the contribution of a driving variable towards explaining the distribution is assigned to another strongly correlated variable. Therefore we present all collinear variables in Table S1.

In the presentation of our results, we are making the assumption that the distribution of environmentally suitable habitats has been approximately stable since ~8,000 BCE (Before Common Era).

**Supplementary Results 1: Results of the accuracy assessments**

Our accuracy assessment showed that the Hansen *et al.* [24] data inferred tree cover, losses and gains for China with an overall accuracy of around 89-93% (Table S3). These values are similar to the global accuracy statistics reported by the authors themselves [24]. However, there was a small tendency for tree cover to be underestimated (i.e. the omission error rate exceeded the commission error rate), and for losses to be over-emphasized. A tendency to underestimate dense tree cover (≥50% crown cover) and gains and to over-emphasize losses was also present in the Sexton *et al.* [10] data, which had an overall accuracy of around 87-89% (Table S4).

Both the Jaxa PALSAR data and GlobeLand30 on the other hand tended to overestimate tree cover and/or gains (applying the criteria used by these datasets, i.e. here tree cover is defined as area with >10% crown cover and there is no specific height threshold). Almost three quarters of the validation cells that switched from a non-forest to a forest stage in the Jaxa PALSAR data between 2007 and 2010 and for which Hansen *et al.* [24] do not report gains did in fact not have gains. Similarly, almost two thirds of the validation cells classed as ‘forest’ by GlobeLand30 and classed as <10% crown cover by Hansen *et al.* [24] in 2000 (with no gains between 2000 and 2010) did in fact not have crown cover >10%. The overall accuracy of GlobeLand30 (with respect to distinguishing forest/non-forest as defined by their own criteria) was also lower than the Hansen *et al.* maps (Table S5). (Note that we did not conduct an independent evaluation of the Jaxa PALSAR data.)

In order to explore the potential shortcomings of the Hansen *et al.* [24] data we mapped all omission and commission errors. These were randomly distributed across the whole of China and all eco-floristic zones (Fig. S14). However, some of the larger omissions of tree cover affected areas with deciduous forests/plantations with regular snow cover (which can negatively bias metrics such as the NDVI). In addition, over a third of the omissions of larger areas were associated with steep slopes or gullies where shade and viewing angle may have interfered with the detection algorithm.

To quantify this further we explored what types of tree cover and gains were omitted in the Hansen *et al.* [24] data: almost 40% were cells at forest/plantation margins or incremental density gains in existing stands. Another third were trees planted in strips or small areas of <5 ha. Eight percent were medium areas of 5-100 ha. The remainder were areas >100 ha, but more than half of these had tree cover <50% and/or consisted of small trees, which could be just at the 5 m height threshold. Thus, while the validation exercises indicated that caution is needed when inferring lack of tree cover and/or gains, these omissions mainly affect small areas with sparse and/or low height tree cover. The higher the canopy cover, the more accurately it could be inferred. According to the data originators the detection algorithm reliably captures stands with >30% canopy cover (= light intercepted by the canopy) >5 m height (Hansen, *pers. com.*). In this paper we consequently focus on large contiguous blocs of tall trees with crown cover ≥50%. For this type of ‘high-quality’ tree cover the data seem solid.

An important question is whether the fact that Hansen *et al.* [24] report few tree cover gains for China could simply be due to data inaccuracies and biases. To explore this we conducted a sensitivity analysis focusing on areas >5 ha with ≥50% canopy cover:

Of the reported gains 9% were in fact no gain. At the same time 3.2% of the validation cells classed by Hansen *et al.* [24] as having no tree cover or no gains had in fact >5 ha (gains of) forest/plantations with canopy cover ≥50%. We estimate that the potential gains of large and dense forest cover range between 15,573 km^2^ (gains minus 9%) and 198,532 km^2^ (gains plus potentially undetected gains). Potentially undetected gains were calculated as follows: (1) we mapped the entire climate space within which omission have occurred using a Species Distribution Modelling approach. (2) To provide a conservative estimate we used a low probability threshold for including area into this space. (3) We then subtracted the area already covered by dense tree cover from this space, and calculated 3.2% of the remainder. The climate space has been established using a MaxEnt model of the 132 omissions combined with the environmental surfaces summarized in Table S1. The ‘hurdle’ that cells would have to pass to be included into this climate space has been selected to be very low (logistic threshold of 0.1).

Of the reported losses 9% were in fact no loss while 5% of losses remained undetected. This bounds potential losses between 45,834 km^2^ (losses minus 9%) and 116,051 km^2^ (losses plus potentially undetected losses calculated as 5% of the area with ≥50% crown cover).

Thus, the potential net changes of large continuous blocs of mature and dense forest are bound between a maximum potential net loss of 100,478 km^2^ and a maximum potential net gain of 152,698 km^2^. The latter corresponds to less than half of the net gains reported by China (Table S2). The large error bounds serve to illustrate that it is enormously challenging to monitor forest cover change in a country as large and heterogeneous as China.

**Supplementary Results 2: Comparison of different forest cover definitions and statistics**

Table S2 summarizes the currently most widely used datasets for estimating forest areas and forest area change globally and/or in China. The estimates vary substantially across the different datasets. For instance, while Hansen *et al.* [24] report a net tree cover loss between for China 2000 and 2012, and Sexton *et al.* [10] record only modest gains, both the China National Forest Inventories and the Chinese forest cover statistics provided to the FAO report significant increases of over 300,000 km^2^.

The differences between these datasets are to a large extent consistent with the respectively applied definitions of ‘forest’. The FAO uses the broadest definition. ‘Forest’ according to the FAO includes all areas under actual and potential tree cover >10%, including immature plantations and bare areas that are to be afforested. China’s National Forest Inventories use a crown cover threshold of ≥20%, but there is no explicit height threshold, and since 2004 ‘special purpose shrubs’ are also classed as forest [33]. Consequently, there is not necessarily a conflict between the large gains reported by China and the modest gains detected by Sexton *et al.* using a 50% crown cover threshold and a height threshold of >5 m. Nor is there a conflict between the gains reported by all of these datasets and the net losses reported by Hansen *et al.* as these latter data only register substantial changes (from ≥50% to ~0% crown cover and *vice versa*), and not gradual changes (growth and degradation). (Note that vegetation height is difficult to infer from remotely sensed imagery unless the classification algorithm is trained using high-resolution LIDAR or field data, i.e. the crown cover threshold is more informative when comparing these datasets.)

For equivalent definitions the datasets tend to produce relatively similar results. The estimates provided by Hansen *et al.* for 20% canopy cover and by the National Forest Inventories for 2000-2003 are approximately equal (within <10,000 km^2^ of each other). The 2005 China National Landcover dataset also produces comparable estimates. Thus the estimates provided by the different datasets for early in the millennium are broadly similar and consistent with differences in the definition of forest. The same is true for forest cover estimates around 2010 for which there are more datasets: one of the highest estimates for tree cover area in China (~2.3 Million km^2^) is provided by China’s National Landcover Dataset, which has a 10% crown cover threshold and includes bush cover. Other datasets employing a 10% crown cover but excluding bush cover all report around 2 Million km^2^. Conversely, the MCD12Q1 data register only 1.7 Million km^2^ with a crown cover threshold of >60% over a reference area of 500 × 500 m and a height threshold of >2 m. The lowest estimate (1.3 Million km^2^) is provided by Hansen *et al.* with height and crown cover thresholds of >5 m and ≥50%. Qin et al. [34] have disaggregated China’s total forest cover according to multiple datasets into different canopy cover classes. Their results show that when only considering dense forest cover (in this case ≥40%) the 2010 forest cover estimates across the various different datasets are more similar to that of Hansen *et al.* and MCD12Q1.

In addition to definitions, discrepancies are likely to arise due to differences in scale-related detection ability. Hansen *et al.* data have a tendency to under-record small and fragmented areas of tree cover (Supplementary Results 1). Similarly, the relatively coarser scale of the MCD12Q1 data (500 m) means that forest areas need to be larger in order to be detected. The same may be true for the Sexton *et al.* data due to the medium resolution of the underlying MODIS data (see Supplementary Methods 1). GlobeLand30 and the Jaxa PALSAR data on the other hand have a tendency to overestimate tree cover (Supplementary Results 1).

In summary, the observed differences in the various datasets are consistent with the notion that the extensive gains claimed by China may include a large amount of low-height, sparse and/or scattered forests (as opposed to large-size, tall and closed forest). This could be due to planting being around small woodlots, narrow strips, shrubs or very slowly maturing trees. However, it is also possible that planting has not always been successful.

**Supplementary Results 3: Comparison of reported afforestation and detectable gains**

According to the statistical yearbooks China planted a total of 1.17 million km^2^ of tree cover between 1992 and 2013. This is an average of 55,000 km^2^ per year (56,000 km^2^ in the 1990s and 55,000 km^2^ since 2000). Tree planting has peaked in 2003 and in any given year has never been less than 25,000 km^2^ (Fig. S11a). The afforestation pattern has been similar across all of China’s regions (Fig. S11b).

There is no correlation between the reported afforestation per province (for any time period) and detectable tree cover gains according to Hansen *et al.* [24] (Fig. S15). While reported afforestation has been highest in North, central and Southwest China the gains detected by Hansen *et al.* were highest in Southeast and Northeast China (Figs. S12 and 15). There is a correlation between the more gradual gains recorded by Sexton *et al.* [10] and reported afforestation (Fig. S15). This is particularly evident when including afforestation data from the 1990s. The correlation is partly driven by larger provinces that have received a lot of afforestation effort such as Heilongjiang, and there is less or no correlation when the figures are standardized for area. These results highlight that greater planting efforts are associated with greater returns, but that these returns may be slow and/or consist of a scarcer canopy cover.

**Supplementary Results 4: Trends in “Intact Forest Landscapes”**

Between 2000 and 2013 China has experienced a loss of 11% (9,883 km^2^) of its intact forest landscapes – defined as “unbroken expanses of natural ecosystems within areas of current forest extent, without signs of significant human activity, and having an area of at least 500 km^2^ [35]. These large forest ecosystems are mainly located in Tibet, Inner Mongolia, Sichuan and Yunnan Provinces. All of these have experienced losses, most notably Yunnan and Sichuan (Fig. S13). The remaining 81,062 km^2^ of intact forests are only partly covered by protected areas of IUCN categories I-V. In particular, large areas in Tibet appear unreserved.

**Table S1**. Environmental variables included in the final tree cover habitat suitability model and their Pearson correlations

| **Variable with percent contribution and permutation importance (scaled to sum 100)** | | **2** | **3** | **4** | **5** | **6** | **7** | **8** | **9** | **10** | **11** | **12** | **13** | **14** | **15** | **16** | **17** | **18** | **19** | **20** | **21** | **22** | **23** | **24** | **25** |
| --- | --- | --- | --- | --- | --- | --- | --- | --- | --- | --- | --- | --- | --- | --- | --- | --- | --- | --- | --- | --- | --- | --- | --- | --- | --- |
| 1. Annual mean temperature (°C) | 3.1% (11.6) | 0.5 | 0.8 | -0.8 | 0.9 | 1.0 | -0.7 | 0.8 | 0.9 | 1.0 | 0.4 | 0.5 | 0.1 | 0.4 | 0.5 | 0.1 | 0.2 | 0.4 | -0.5 | 0.5 | -1.0 | 0.7 | -0.5 | -0.2 | -0.1 |
| 2. Mean diurnal temperature range (°C) | 0.4% (2.2) |  | 0.4 | -0.2 | 0.7 | 0.3 | 0.0 | 0.5 | 0.6 | 0.4 | -0.2 | -0.1 | -0.4 | 0.5 | -0.1 | -0.4 | -0.2 | 0.5 | -0.8 | -0.1 | -0.5 | 0.8 | -0.4 | 0.2 | 0.0 |
| 3. Isothermality (ratio of diurnal to annual temperature range) | 0.1% (0.4) |  |  | -0.9 | 0.6 | 0.9 | -0.8 | 0.6 | 0.7 | 0.9 | 0.6 | 0.6 | 0.2 | 0.3 | 0.6 | 0.3 | 0.4 | 0.2 | -0.2 | 0.7 | -0.8 | 0.5 | -0.5 | 0.0 | 0.0 |
| 4.Temperature seasonality | 0.3% (1.2) |  |  |  | -0.5 | -0.9 | 1.0 | -0.5 | -0.6 | -0.9 | -0.6 | -0.6 | -0.3 | -0.2 | -0.6 | -0.3 | -0.4 | -0.2 | 0.1 | -0.6 | 0.8 | -0.4 | 0.3 | 0.0 | 0.0 |
| 5. Max. temperature warmest month (°C) | 0.5 %  (1) |  |  |  |  | 0.8 | -0.4 | 0.8 | 1.0 | 0.8 | 0.1 | 0.2 | -0.1 | 0.4 | 0.2 | -0.1 | 0.0 | 0.4 | -0.6 | 0.3 | -0.9 | 0.8 | -0.5 | -0.3 | -0.2 |
| 6. Minimum temperature coldest month (°C) | 0 %  (0) |  |  |  |  |  | -0.9 | 0.7 | 0.8 | 1.0 | 0.5 | 0.5 | 0.2 | 0.3 | 0.5 | 0.2 | 0.3 | 0.3 | -0.3 | 0.6 | -1.0 | 0.6 | -0.4 | -0.1 | -0.1 |
| 7. Annual temperature range (°C) | 0.1% (0.6) |  |  |  |  |  |  | -0.4 | -0.5 | -0.8 | -0.6 | -0.6 | -0.4 | -0.1 | -0.6 | -0.4 | -0.4 | -0.1 | 0.0 | -0.6 | 0.7 | -0.3 | 0.2 | 0.0 | -0.1 |
| 8. Mean temperature wettest quarter (°C) | 0.4% (1.2) |  |  |  |  |  |  |  | 0.9 | 0.7 | 0.3 | 0.4 | 0.0 | 0.4 | 0.4 | 0.0 | 0.3 | 0.3 | -0.4 | 0.4 | -0.8 | 0.6 | -0.5 | -0.3 | -0.2 |
| 9. Mean temperature warmest quarter (°C) | 14.6% (17.5) |  |  |  |  |  |  |  |  | 0.8 | 0.2 | 0.3 | -0.1 | 0.4 | 0.3 | 0.0 | 0.1 | 0.4 | -0.6 | 0.4 | -0.9 | 0.8 | -0.5 | -0.3 | -0.2 |
| 10. Mean temperature coldest quarter (°C) | 0.1% (0.1) |  |  |  |  |  |  |  |  |  | 0.5 | 0.5 | 0.1 | 0.3 | 0.5 | 0.2 | 0.3 | 0.3 | -0.4 | 0.6 | -1.0 | 0.6 | -0.4 | -0.1 | -0.1 |
| 11. Annual precipitation (mm) | 0.7% (3.1) |  |  |  |  |  |  |  |  |  |  | 0.9 | 0.7 | -0.2 | 0.9 | 0.8 | 0.8 | -0.3 | 0.5 | 0.9 | -0.4 | -0.2 | -0.3 | -0.1 | 0.1 |
| 12. Precipitation wettest month (mm) | 0%  (0) |  |  |  |  |  |  |  |  |  |  |  | 0.4 | 0.1 | 1.0 | 0.4 | 0.7 | -0.2 | 0.4 | 0.8 | -0.5 | 0.0 | -0.4 | -0.1 | 0.1 |
| 13. Precipitation driest month (mm) | 0.1% (0.2) |  |  |  |  |  |  |  |  |  |  |  |  | -0.5 | 0.4 | 1.0 | 0.6 | -0.3 | 0.5 | 0.5 | -0.1 | -0.3 | 0.0 | -0.1 | 0.1 |
| 14. Precipitation seasonality | 0.6% (3.8) |  |  |  |  |  |  |  |  |  |  |  |  |  | 0.1 | -0.5 | -0.1 | 0.5 | -0.5 | -0.1 | -0.3 | 0.5 | -0.4 | 0.2 | 0.0 |
| 15. Precipitation wettest quarter (mm) | 0.2% (2.5) |  |  |  |  |  |  |  |  |  |  |  |  |  |  | 0.5 | 0.8 | -0.2 | 0.4 | 0.9 | -0.5 | 0.0 | -0.4 | -0.1 | 0.1 |
| 16. Precipitation driest quarter (mm) | 0.9% (1.2) |  |  |  |  |  |  |  |  |  |  |  |  |  |  |  | 0.6 | -0.3 | 0.6 | 0.6 | -0.1 | -0.3 | 0.0 | -0.1 | 0.1 |
| 17. Precipitation warmest quarter (mm) | 0%  (0.2) |  |  |  |  |  |  |  |  |  |  |  |  |  |  |  |  | -0.4 | 0.5 | 0.8 | -0.2 | -0.2 | -0.3 | 0.0 | 0.2 |
| 18. Number of months with <3 mm rainfall/month | 0%  (0) |  |  |  |  |  |  |  |  |  |  |  |  |  |  |  |  |  | -0.6 | -0.3 | -0.3 | 0.6 | -0.2 | 0.1 | -0.1 |
| 19. Water stress (AET/PET) | 29% (25.8) |  |  |  |  |  |  |  |  |  |  |  |  |  |  |  |  |  |  | 0.5 | 0.4 | -0.8 | 0.2 | -0.1 | 0.2 |
| 20. Actual evapotranspiration | 38.2% (10.8) |  |  |  |  |  |  |  |  |  |  |  |  |  |  |  |  |  |  |  | -0.5 | -0.1 | -0.4 | -0.1 | 0.1 |
| 21. Ground frost frequency (days) | 0.5% (2.7) |  |  |  |  |  |  |  |  |  |  |  |  |  |  |  |  |  |  |  |  | -0.7 | 0.5 | 0.2 | 0.1 |
| 22. Monthly mean sunshine hours (% of maximum possible) | 5.3% (4.5) |  |  |  |  |  |  |  |  |  |  |  |  |  |  |  |  |  |  |  |  |  | -0.3 | 0.1 | 0.0 |
| 23. Monthly mean wind speed 10 m above ground (m s^-1^) | 0.8% (1.2) |  |  |  |  |  |  |  |  |  |  |  |  |  |  |  |  |  |  |  |  |  |  | -0.1 | -0.1 |
| 24. Elevation (m) | 1%  (2.6) |  |  |  |  |  |  |  |  |  |  |  |  |  |  |  |  |  |  |  |  |  |  |  | 0.5 |
| 25. Slope (degree) | 2.6% (4.7) |  |  |  |  |  |  |  |  |  |  |  |  |  |  |  |  |  |  |  |  |  |  |  |  |
| 26. Soil type | 0.5% (1.1) | (non-numeric) | | | | | | | | | | | | | | | | | | | | | | | |

**Table S2: Comparison of forest definitions and extent of different data products**

| **Dataset** | **Definition of forest** | **Year** | **Forest (km^2^)** | **Area change (km^2^)** |
| --- | --- | --- | --- | --- |
| China National forest inventories [36] (permanent plots and remotely sensed imagery) | Canopy cover ≥20% (modified from 30% in 1994). Since 2004 includes special purpose scrubs in areas <400 mm yr^-1^ precipitation or above the tree line with canopy cover ≥30%. This modification added 0.39% (c. 40,000 km^2^) to overall forest cover [34]. | 1998 | 1,589,400 | +32,780 yr^-1^  (2003-2013) |
|  |  | 2003 | 1,749,100 |  |
|  |  | 2008 | 1,954,500 |  |
|  |  | 2013 | 2,076,900 |  |
| Hansen *et al.* [24] (Landsat) | All vegetation >5 m in height. (Change is switches from 0-≥50% and ≥50-0% canopy cover.) | 2000 | 1,890,162 ≥10% 1,755,489 ≥20% 1,313,676 ≥50% | -2,807 yr^-1^ ≥50% (2000-2012) |
| Sexton *et al.* [10] (MODIS and Landsat) | All vegetation >5 m in height. | 2000  2010 | 925,475 ≥50%  958,439 ≥50% | +3,296 yr^-1^ ≥50% (2000-2010) |
| China National Landcover Dataset [26, 34, 37] (visual interpretation of Landsat imagery) | Forest or plantations with canopy cover >10%, nurseries, forest after logging and orchards. Young plantations that are not detectable from remotely sensed imagery are excluded. | 2005  2010 | 1,756,512 (excl. bush)  2,246,273 (incl. bush)  2,270,000 (incl. bush) | +4,745 yr^-1^ incl. bush (2005-2010) |
| FAO [38, 39] (country reports) | Canopy cover (or equivalent stocking level) >10% and area >0.5 ha. Trees should be able to reach >5 m at maturity. Includes young stands that yet have to reach the criteria, and temporarily unstocked areas that are expected to revert to forest. | 2000  2010 | 1,634,800  2,068,610 | +43,391 yr^-1^ (2000-2010) |
| GlobeLand30 (National Geomatics Center of China) [28] (Landsat) | Land covered with trees, with vegetation cover >30%, including deciduous and coniferous forests, and sparse woodland with cover 10-30%. | 2010 | 2,144,187 | - |
| Jaxa PALSAR-2/PALSAR 25 m Forest/Non-forest [27] | Canopy cover >10% and area >0.5 ha. | 2010 | 1,945,000 | - |
| PALSAR MODIS 50 m Forest/Non-forest for China [34] | Woody vegetation with canopy >10%. | 2010 | 2,020,000 | - |
| IGBP MODIS MCD12Q1 Forest/Non-forest map [40] 500 m | Woody vegetation with canopy >60% and >2 m height. | 2010 | 1,686,000 | - |
| ESA MERIS and SPOT land cover map [41] 300 m | Woody vegetation with canopy >15%. | ~ 2010 | 1,940,100 | - |

**Table S3. Accuracy assessment of the Hansen *et al.* [24] maps of recent tree cover change**

a) Unchanged tree cover in three categories (n = 685)

|  | Reference |  |  |  |  |  |  |
| --- | --- | --- | --- | --- | --- | --- | --- |
| Map |  | No tree cover | Tree cover 1-<50% | Tree cover ≥50% | Total | User’s accuracy (SE) | 95% CI |
| No tree cover | | 182 | 26 | 9 | 217 | 83.9 (2.5) | 78.7 – 89 |
| Tree cover 1-<50% | | 14 | 219 | 17 | 250 | 87.6 (2.1) | 83.3 – 91.9 |
| Tree cover ≥50% | | 2 | 18 | 198 | 218 | 90.8 (2) | 86.8 – 94.9 |
| Total | | 198 | 263 | 224 | 685 |  |  |
| Producer’s accuracy (SE) | | 91.9 (1.9) | 83.3 (2.3) | 88.4 (2.1) |  |  |  |
| 95% CI | | 87.9 – 96 | 78.6 – 88 | 84 – 92.8 |  |  |  |
| Overall accuracy = 88.6 (84.9 – 90); kappa = 81.1 (77.3 – 84.9) | | | | | | |  |

b) Unchanged tree cover in two categories (n = 685)

|  | Reference |  |  |  |  |  |
| --- | --- | --- | --- | --- | --- | --- |
| Map |  | Tree cover 0-<50% | Tree cover  ≥50% | Total | User’s accuracy (SE) | 95% CI |
| Tree cover 0-<50% | | 441 | 26 | 467 | 94.4 (1.1) | 92.2 – 96.6 |
| Tree cover ≥50% | | 20 | 198 | 218 | 90.8 (2) | 86.8 – 94.9 |
| Total | | 461 | 224 | 685 |  |  |
| Producer’s accuracy (SE) | | 95.7 (0.9) | 88.4 (2.1) |  |  |  |
| 95% CI | | 93.7 – 97.6 | 84 – 92.8 |  |  |  |
| Overall accuracy = 93.3 (91.3 – 95.2); kappa = 84.6 (80.3 – 89) | | | | | | |

c) Loss 2000 - 2012 (n = 400)

|  | Reference | |  |  |  |  |  |
| --- | --- | --- | --- | --- | --- | --- | --- |
| Map |  | | Loss | No loss | Total | User’s accuracy (SE) | 95% CI |
| Loss | | | 182 | 18 | 200 | 91 (2) | 86.8 – 95.2 |
| No loss | | | 11 | 189 | 200 | 94.5 (1.6) | 91.1 – 97.9 |
| Total | | | 193 | 207 | 400 |  |  |
| Producer’s accuracy (SE) | | | 95.3 (1.7) | 91.3 (2) |  |  |  |
| 95% CI | | | 90.8 – 97.8 | 87.2 – 95.4 |  |  |  |
| Overall accuracy = 92.6 (90.1 – 95.4); kappa = 85.5 (80.3 – 90.7) | | | | | | | |
|  | |  | | | | | |
|  | |  | | | | | |

d) Gain 2000 - 2012 (n = 400)

|  | Reference |  |  |  |  |  |
| --- | --- | --- | --- | --- | --- | --- |
| Map |  | Gain | No gain | Total | User’s accuracy (SE) | 95% CI |
| Gain | | 181 | 19 | 200 | 90.5 (2.1) | 86.2 – 94.8 |
| No gain | | 22 | 178 | 200 | 89 (2.2) | 84.4 – 93.6 |
| Total | | 203 | 197 | 400 |  |  |
| Producer’s accuracy (SE) | | 89.2 (2.2) | 90.4 (2.1) |  |  |  |
| 95% CI | | 84.6 – 93.7 | 86 – 94.7 |  |  |  |
| Overall accuracy = 89.8 (86.7 – 92.9); kappa = 79.5 (73.4 – 85.6) | | | | | | |

**Table S4: Accuracy assessment for the Sexton *et al.* [10] maps of tree cover and change**

a) Unchanged tree cover in two categories (n = 665)

|  | Reference | |  |  |  |  |  |
| --- | --- | --- | --- | --- | --- | --- | --- |
| Map |  | | Tree cover 0-<50% | Tree cover  ≥50% | Total | User’s accuracy (SE) | 95% CI |
| Tree cover 0-<50% | | | 446 | 57 | 503 | 88.7 (1.4) | 85.8 – 91.5 |
| Tree cover ≥50% | | | 27 | 135 | 162 | 83.3 (2.9) | 77.3 – 89.4 |
| Total | | | 473 | 192 | 665 |  |  |
| Producer’s accuracy (SE) | | | 94.3 (1.1) | 70.3 (3.3) |  |  |  |
| 95% CI | | | 92.1 – 96.5 | 63.6 – 77 |  |  |  |
| Overall accuracy = 87.4 (84.8 – 90); kappa = 67.8 (61.3 – 74.2) | | | | | | | |
|  | |  | | | | | |

**b) Loss 2000 - 2010 (n = 133**)

|  | Reference |  |  |  |  |  |
| --- | --- | --- | --- | --- | --- | --- |
| Map |  | Loss | No loss | Total | User’s accuracy (SE) | 95% CI |
| Loss | | 35 | 14 | 49 | 71.4 (6.5) | 57.8 – 85.1 |
| No loss | | 1 | 83 | 84 | 98.8 (1.2) | 85.1 – 100 |
| Total | | 36 | 97 | 133 |  |  |
| Producer’s accuracy (SE) | | 97.2 (2.7) | 85.6 (3.6) |  |  |  |
| 95% CI | | 90.5 – 100 | 78.1 – 93.1 |  |  |  |
| Overall accuracy = 88.7 (83 – 94.5); kappa = 74.4 (62.1 – 86.6) | | | | | | |

**c**) Gain 2000 - 2010 (n = 197)

|  | Reference |  |  |  |  |  |
| --- | --- | --- | --- | --- | --- | --- |
| Map |  | Gain | No gain | Total | User’s accuracy (SE) | 95% CI |
| Gain | | 55 | 2 | 57 | 96.5 (2.4) | 90.8 – 100 |
| No gain | | 20 | 120 | 140 | 85.7 (3) | 79.6 – 91.9 |
| Total | | 75 | 122 | 197 |  |  |
| Producer’s accuracy (SE) | | 73.3 (5.1) | 98.4 (1.1) |  |  |  |
| 95% CI | | 62.7 – 84 | 95.7 – 100 |  |  |  |
| Overall accuracy = 88.8 (84.2 – 93.5); kappa = 75.2 (65.4 – 85) | | | | | | |

Table S5: Accuracy assessment for GlobeLand30 2010 forest/non-forest maps (n=500)

|  | Reference | |  |  |  |  |  |
| --- | --- | --- | --- | --- | --- | --- | --- |
| Map |  | | No forest | Forest | Total | User’s accuracy (SE) | 95% CI |
| No forest | | | 261 | 39 | 300 | 87 (1.9) | 83 – 91 |
| Forest | | | 34 | 166 | 200 | 83 (2.7) | 77.5 – 88.5 |
| Total | | | 295 | 205 | 500 |  |  |
| Producer’s accuracy (SE) | | | 88.5 (1.9) | 81 (2.7) |  |  |  |
| 95% CI | | | 84.7 – 92.3 | 75.4 – 86.6 |  |  |  |
| Overall accuracy = 85.4 (82.2 – 88.6); kappa = 69.7 (63.2 – 76.2) | | | | | | | |
|  | |  | | | | | |

**Figure S1. Tree cover gains and losses in China between 2000 and 2010 according to Sexton *et al.* [10]. Losses (red) and gains (blue) of tree cover with ≥50% canopy cover. The net gain measured with these data is 32,964 km^2^.**

|  |  |
| --- | --- |
|  |  |

**Figure S2. More details on the data generated by Sexton *et al.* [10].** (a) Histogram of the magnitude and direction of crown cover change across China in 30 × 30 m cells. (b) Count of image acquisitions across months (from January = 1 to December = 12, x axis) for 2000 and (c) 2010. Image assignment to climate zones: P134-129, R47-40: tropical dry, growing season Jul-Dec; P128-118, R47-40: tropical dry, growing season May-Dec; rest: growing season May-Oct.

**a**


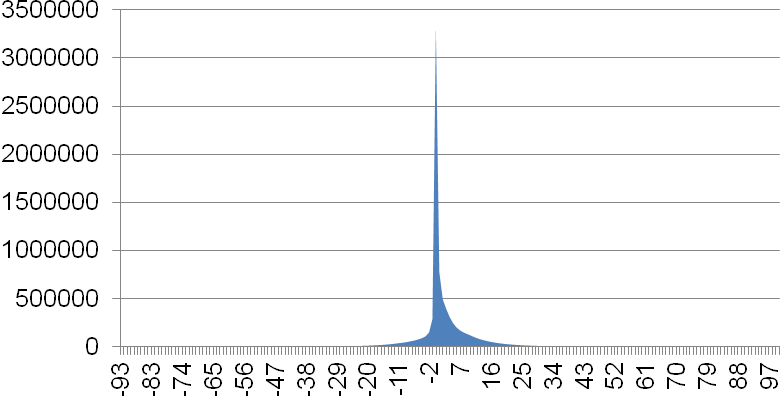


**Km^2^**

**Percentage change in crown cover**

**
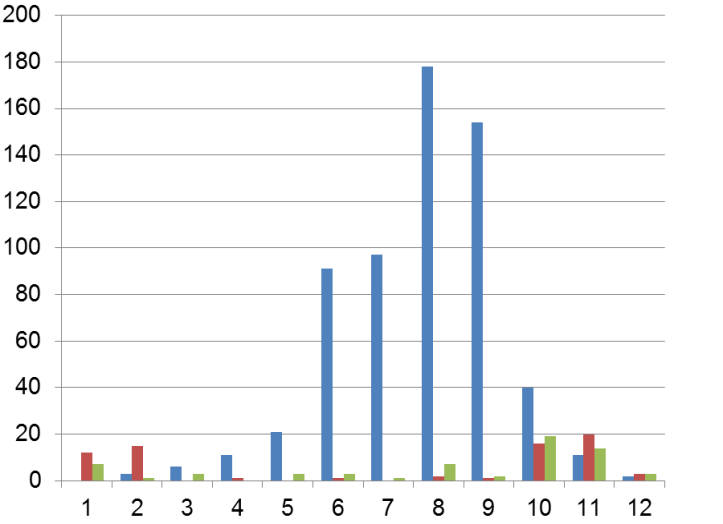

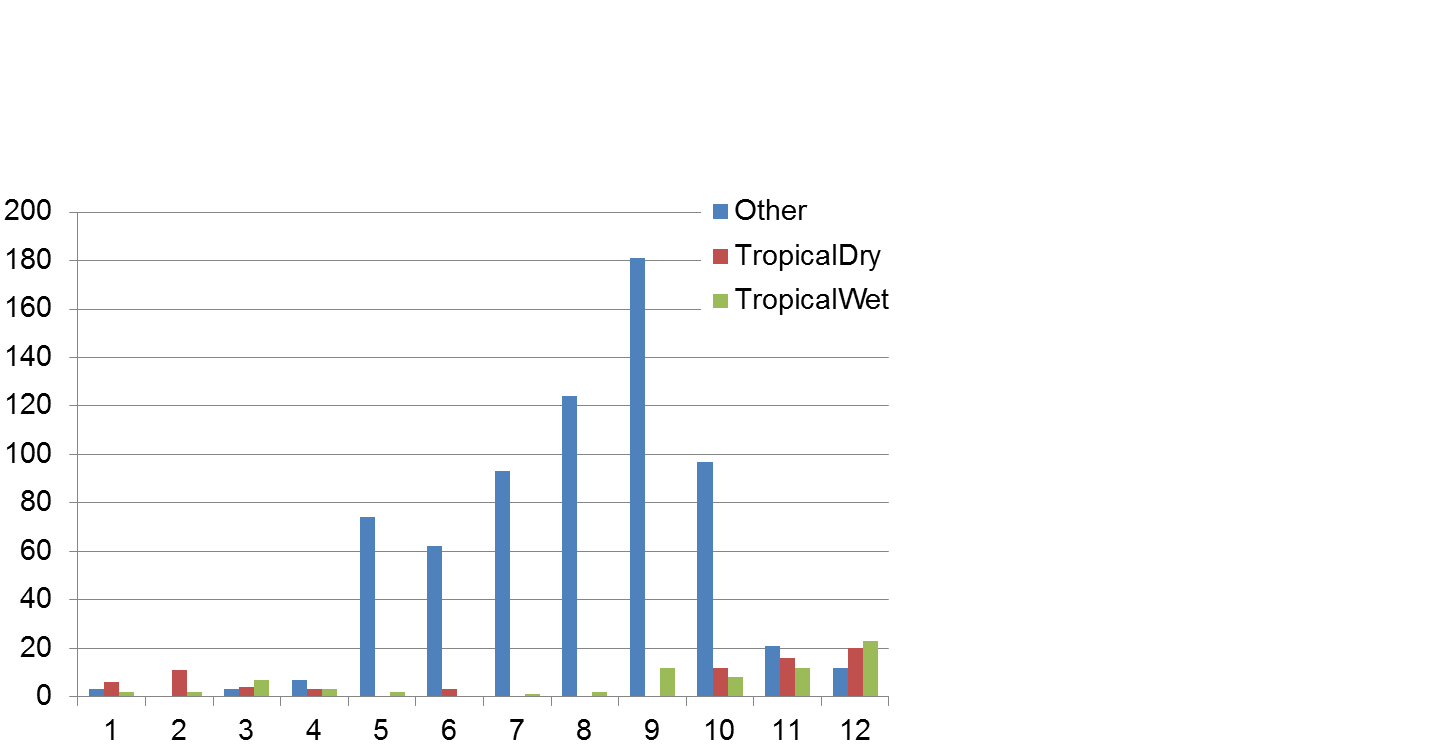
**

**b**

**c**

**Month**

**Image acquisitions 2000**

**Image acquisitions 2010**

**Month**

**Figure S3. Summary of forest area figures for China provided by different datasets with different definitions of forest.** (a) Forest area figures for ~2000 and ~2010. (b) Annual forest area change. For more details see Table S2.

| **a** | 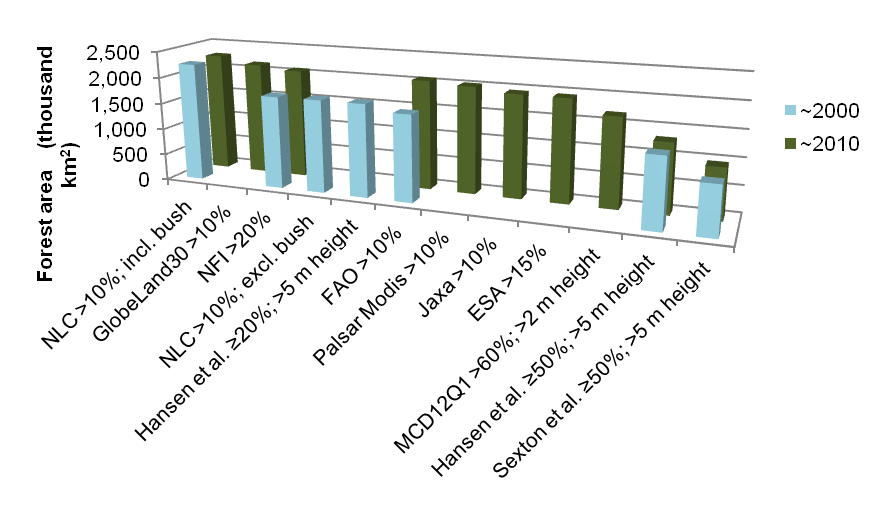 |  |
| --- | --- | --- |
|  | **Details on the datasets and their definitions of forest/tree cover (for further details and references see Supplementary Results 2 and Table S2):** (1) NLC - China National Landcover Dataset: canopy cover (cc) >10%, includes bush cover; resolution (res) 30 m; here for 2005 and 2010. (2) GlobeLand30: cc >10%; res 30 m; 2000 and 2010. (3) NFI – China National Forest Inventory: cc ≥20%; includes special purpose scrubs with cc ≥30%; 2003 and 2013. (4) Hansen *et al.*: cc ≥20% and ≥50%; tree height >5 m; res 30 m but according to our validation robust for c. 225 m; 2000 and 2012. (5) FAO - cc >10%; tree height ≥5 m at maturity; res c. 71 m; includes young stands and temporarily unstocked areas; 2000 and 2010. (6) Palsar Modis - PALSAR MODIS Forest/Non-forest for China: cc >10%; res 50 m; 2010. (7) Jaxa - Jaxa PALSAR-2/PALSAR 25 m Forest/Non-forest: cc >10%; res 25 m; 2010. (8) ESA - ESA MERIS and SPOT land cover map: cc >10%; res 300 m; 2010. (9) MCD12Q1 - IGBP MODIS MCD12Q1 Forest/Non-forest map: cc >60%; tree height >2 m; res 500 m; 2010. (10) Sexton *et al.*: cc ≥50%; tree height >5 m; res 30 m but according to our validation robust for c. 225 m; 2000 and 2010. (The data may have a tendency to underestimate high levels of canopy cover but this bias is consistent in 2000 and 2010 – see Supplementary Methods 1.) |  |
| **b** | 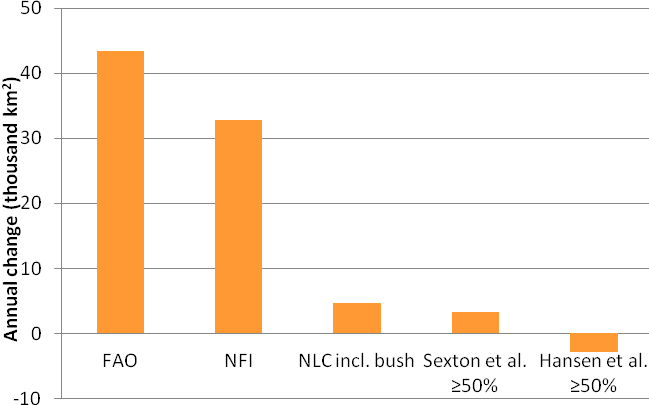 |  |

**Figure S4.** **Global tree cover changes across altitude, slope, nearest city growth rate, and protection status.** The analysis is restricted to tree cover ≥50% and based on data generated by Hansen *et al.* [24]. The percentages show total remaining tree cover in 2012 (mean modelled remaining tree cover + recent gains), and the error bars indicate the range of predictions of the tree cover suitability models. For associated maps see Fig. S10. Recent tree cover losses ranged from 0.8 Mio km^2^ to 0.15 Mio km^2^ across the altitude classes (low to high), and from 1.15 Mio km^2^ to almost zero across slope (low to high). Losses from unprotected areas totalled 1.75 Mio km^2^ (5.3%), and from within protected areas 0.09 Mio km^2^ (2.9%). With respect to city growth, recent tree cover losses were highest (0.72 Mio km^2^) in the vicinity of cities with a medium growth rate. For other categories presented in Fig. 3: Recent tree cover losses were highest in the tropics (0.81 Mio km^2^), followed by the boreal regions (0.43 Mio km^2^). Regarding population pressures, recent tree cover losses across the population density classes (high to low) ranged from 0.53 Mio km^2^ to 0.21 Mio km^2^; across distances to towns (low to high) from 0.52 Mio km^2^ to 0.18 Mio km^2^; and across agricultural suitability (high to low) from 0.91 Mio km^2^ to 0.28 Mio km^2^.

**Figure S5.** **Tree cover changes in low income countries (GDP per capita <US$ 10,000).** Recent tree cover losses ranged from 318,000 km^2^ to 5,000 km^2^ across the population density classes (high to low); from 249,000 km^2^ to 12,000 km^2^ across distances to towns (low to high); from 163,000 km^2^ to 30,000 km^2^ across agricultural suitability (high to low); from 279,000 km^2^ to 58,000 km^2^ across altitude (low to high); and from 340,000 km^2^ to 7,000 km^2^ across slope (low to high). With respect to city growth, recent tree cover losses were highest (218,000 km^2^) in the vicinity of cities with a medium growth rate. Recent losses from unprotected areas totalled 497,000 km^2^ (4%), and 18,000 km^2^ (2%) from within protected areas. For further explanations see legend Fig. S4 and for associated maps see Fig. S10. The underlying data has been generated by Hansen *et al.* [24]. Note that almost 70% of the total area of low income countries is located in the tropics.

**Figure S6.** **Tree cover changes in high income countries (GDP per capita ≥US$ 10,000).** Recent tree cover losses were highest at low altitudes (520,000 km^2^), shallow slopes (812,000 km^2^) and in non-protected areas (1.08 Mio km^2^; 6%). With respect to population pressures the pattern was less clear than in low-income areas. Recent tree cover losses also affected areas with low population density, at large distances from towns and with low agricultural suitability. This pattern is mostly driven by boreal forests where tree cover losses are frequently related to natural causes. For further explanations see legend Fig. S4 and for associated maps see Fig. S10. The underlying data has been generated by Hansen *et al.* [24].

**Figure S7.** **Proportionate net tree cover loss by country.** (a) Proportionate net losses against annual population growth (*F* = 52, *df* = 116, *R^2^* = 0.5, *p*≤0.001). (b) Proportionate net losses against urbanization rate (*F* = 38, *df* = 116, *R^2^* = 0.4, *p*≤0.001). (c) Proportionate net losses against the percent contribution of agriculture to the GDP (*F* = 19, *df* = 102, *R^2^* = 0.3, *p*≤0.001). (d) Proportionate net losses against food production increase (*F* = 14, *df* = 117, *R^2^* = 0.2, *p*≤0.001). The trend line is based on natural cubic splines and the grey shading shows the 95% Confidence Intervals. The analysis is restricted to countries with ≥10,000 km^2^ tree cover in 2000. Exemplar countries are listed. The underlying data has been generated by Hansen *et al.* [24].

**c**

**a**

**d**

**b**

**Figure S8.** **Principal Components Analysis (PCA) of macroeconomic indicators (a), and the position of individual countries in terms of tree cover loss (b).** The PCA is centred and scaled to unit variance. The country names are colour coded according to their recent (2000 – 2012) proportionate net tree cover loss from high (red) to low (green). There is a clear signal that countries with high levels of poverty, urbanisation and population growth have also experienced the highest levels of uncompensated tree cover loss (first axis). The second axis shows that tree cover losses also tend to be high in countries which have low amounts of tree cover per person, a large proportion of tree cover in close proximity to towns and/or areas with high population density and high agricultural suitability.


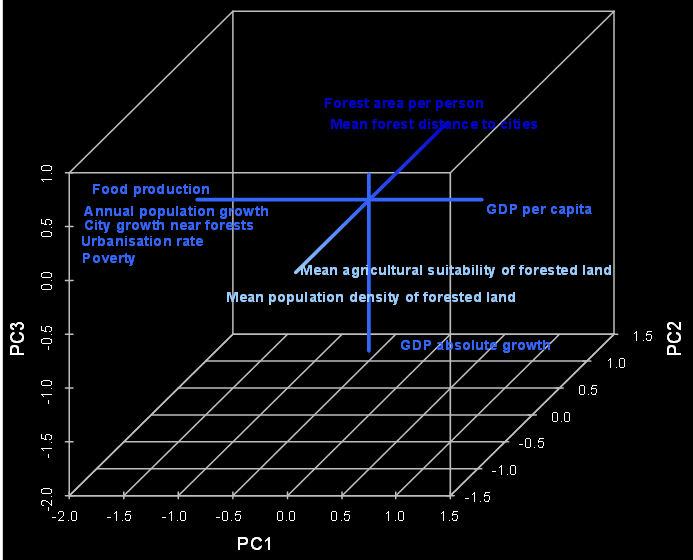


**a**

**b**

**Figure S9.** **Tree cover changes in China across altitude, slope, nearest city growth rate, and protection status.** Recent tree cover losses ranged from 15,000 km^2^ to 8,000 km^2^ across the altitude classes (low to high), and they predominantly affected slopes of >1-10° (40,000 km^2^). Losses from unprotected areas totalled 47,000 km^2^ (3.8%), and from within protected areas 3,000 km^2^ (3.2%). With respect to city growth, recent tree cover losses were highest (21,000 km^2^) in the vicinity of cities with a medium growth rate. For other categories presented in Fig. 3: Recent tree cover losses were highest in the subtropics (38,000 km^2^). Regarding population pressures, recent tree cover losses across the population density classes (high to low) ranged from 45,000 km^2^ to almost zero; across distances to towns (low to high) from 41,000 km^2^ to 3,000 km^2^; and across agricultural suitability (high to low) from 29,000 km^2^ to almost zero. For further explanations see legend Fig. S4. The underlying data has been generated by Hansen *et al.* [24].

**Figure S10.** **Global population pressure/accessibility maps.** These maps accompany Figs. 3, S4-6 and S9. Data sources in Methods.

**
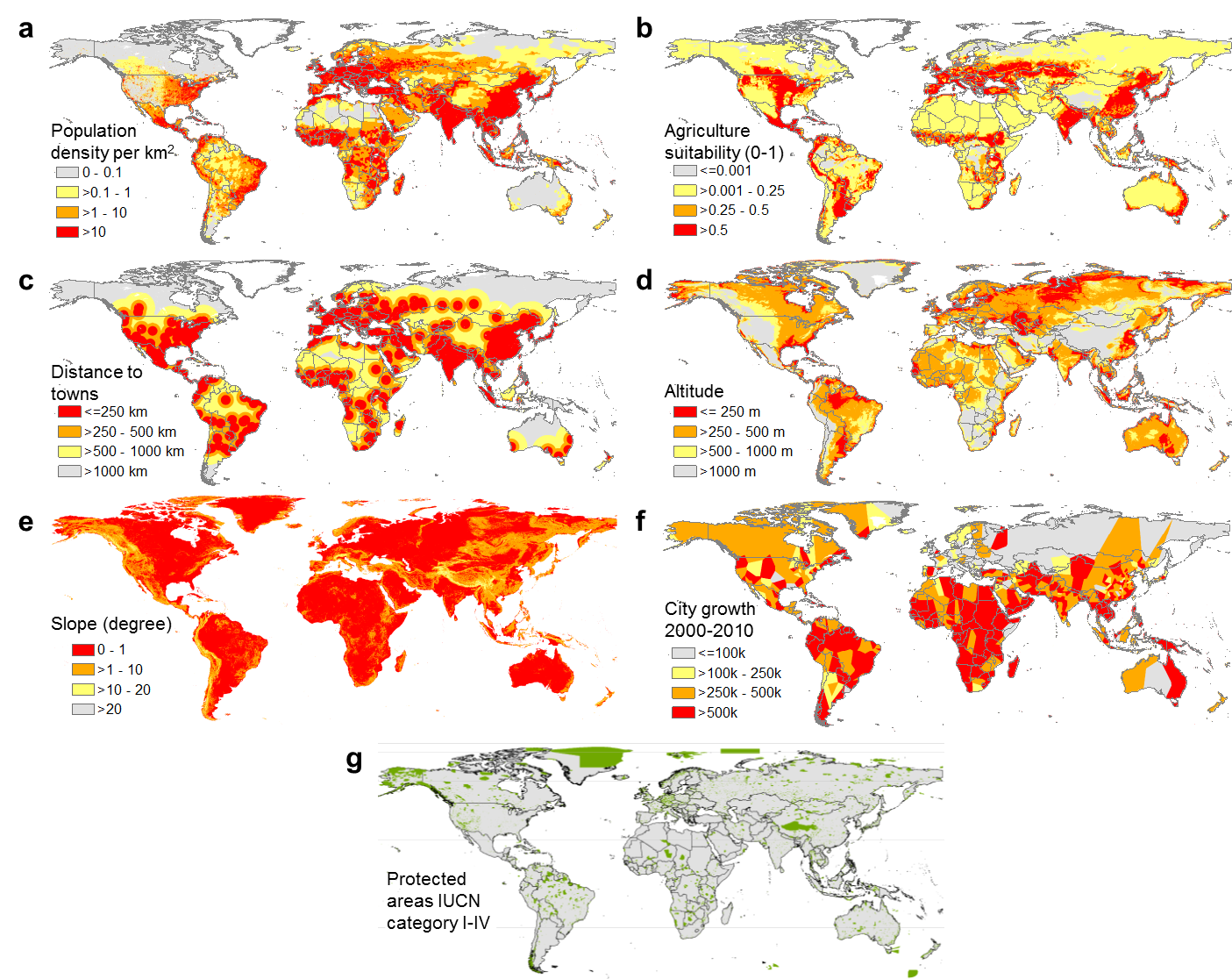
**

**Figure S11. Afforestation statistics according to China’s Statistical Yearbooks.** (a) Reported afforestation across China. (b) Reported afforestation by Region.

**a**


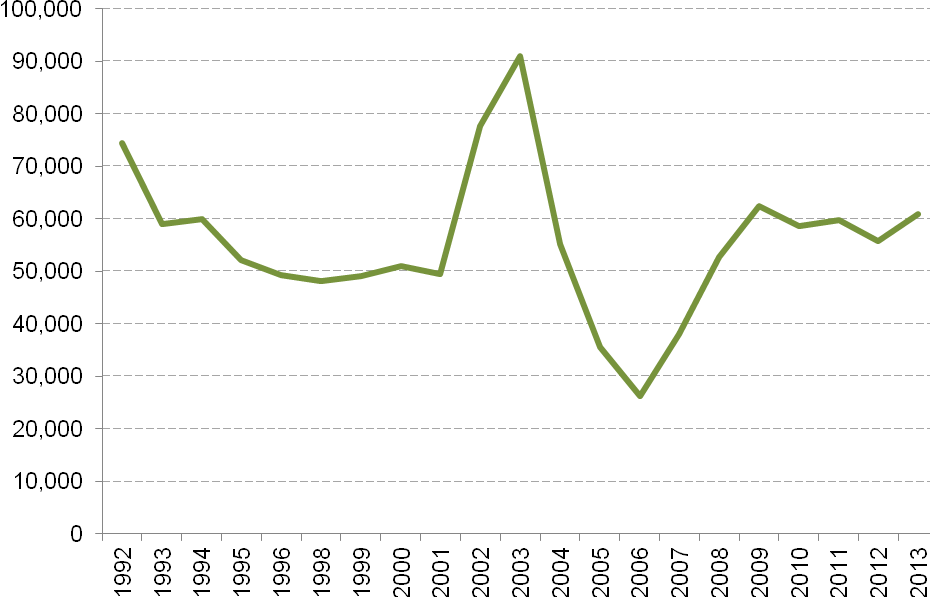


**Km^2^**

**Year**

**b**


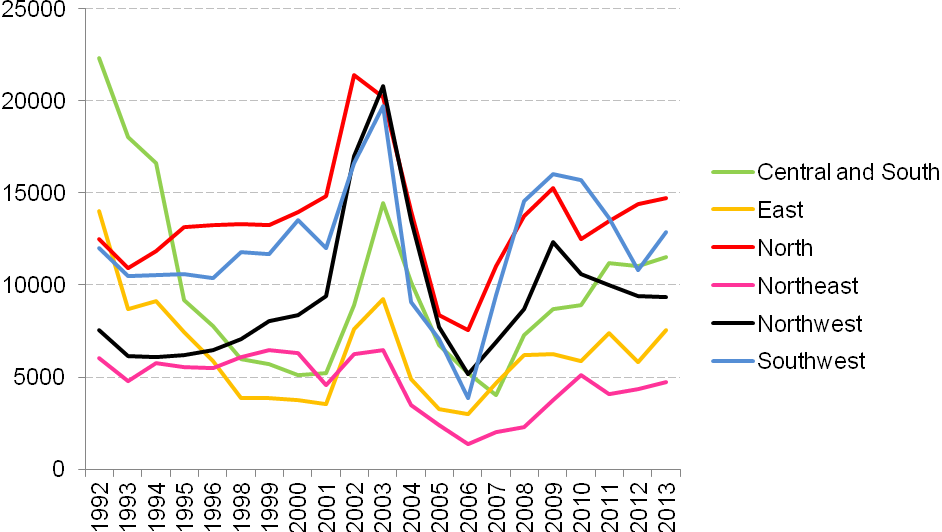


**Km^2^**

**Year**

**Figure S12. Reported afforestation and detectable gains in China.** (a) Reported afforestation 1992-2012 (km^2^). (b) Reported afforestation 1992-2012 as percentage of province area. (c) Reported afforestation 2000-2012 (km^2^). (d) Reported afforestation 2000-2012 as percentage of province area. (e) Gains according to Hansen *et al.* [25] (km^2^). (f) Gains according to Hansen *et al.* [24] as percentage of province area. (g) Gains of canopy cover ≥50% according to Sexton *et al.* [10] (km^2^). (h) Gains of canopy cover ≥50% according to Sexton *et al.* [10] as percentage of province area.

| **a**  **b**  **Km^2^** |  |
| --- | --- |
| **c**  **d**  **Km^2^** |  |
| **e**  **f**  **Km^2^** |  |

**g**

**h**

**Km^2^**

**Figure S13. Trends in intact forest landscapes over China [35, 42]**. Between 2000 and 2013 China has lost 9,883 km^2^ of intact forest landscapes to now 81,062 km^2^.

|  |
| --- |

**Figure S14.** **Validation maps for the data generated by Hansen *et al.* [24].** (a) Validation of tree cover estimates across three categories: no tree cover (n=217 + n=200 contentious data points), tree cover ≤50% (n=250) and tree cover ≥50% (n=218). Red: tree cover underestimated; yellow: tree cover overestimated; black: correct values. (b) Validation of losses (n=400). Commissions are in red and omission in green. (c) Validation of gains (n=400). Commissions are in green and omission in red. (d) Combined map of all omissions of tree cover and gains detected during the validation (n=146). Small dots: areas <5 ha; medium sized dots: areas 5-100 ha; large dots: areas >100 ha.

| **a** | **b** |
| --- | --- |
| **c** | **d** |

**Figure S15.** Correlations between reported afforestation and detectable gains

| **a** | **Detectable gains** |  |
| --- | --- | --- |
|  |  |
| **b** |  |
|  |  | **Reported afforestation** |

**Supplementary references**

1. Sun C., Chen L. 2006 A study of policies and legislation affecting payments for watershed services in China. (ed. Research Centre of Ecological and Environmental Economics Beijing and the International Institute for Environment and Development). London.

2. Cao S., Chen L., Shankman D., Wang C., Wang X., Zhang H. 2011 Excessive reliance on afforestation in China's arid and semi-arid regions: Lessons in ecological restoration. *Earth-Sci Rev* **104**(4), 240-245. (doi:10.1016/j.earscirev.2010.11.002).

3. State Forestry Administration China. 2011 China National Progress Report to the UNFF Secretariat on the implementation of NLBI and other relevant resolutions. (ed. State Forestry Administration China). Beijing.

4. Delang C.O., Yuan Z. 2015 *China’s Grain for Green Program*. Dordrecht, Springer.

5. Dai L., Zhao W., Shao G., Lewis B.J., Yu D., Zhou L., Zhou W. 2013 The progress and challenges in sustainable forestry development in China. *Int J Sust Dev World* **20**(5), 394-403.

6. Ren G., Young S.S., Wang L., Wang W., Long Y., Wu R., Li J., Zhu J., Douglas W.Y. 2015 Effectiveness of China's National Forest Protection Program and nature reserves. *Conserv Biol* **29**(5), 1368-1377. (doi:10.1111/cobi.12561).

7. DiMiceli C.M., Carroll M.L., Sohlberg R.A., Huang C., Hansen M.C., Townshend. J.R.G. 2011 *Annual Global Automated MODIS Vegetation Continuous Fields (MOD44B) at 250 m Spatial Resolution for Data Years Beginning Day 65, 2000-2010, Collection 5 Percent Tree Cover.* University of Maryland. http://www.landcover.org/data/vcf/. Accessed January 2016.

8. Gutman G., Byrnes R., Covington M., Justice C., Franks S., Headley R. 2005 Towards monitoring land-cover and land-use changes at global scale: The global land use survey. *Photogramm Eng Remote Sensing* **64**, 6-10.

9. R Core Team. 2017 *R: A language and environment for statistical computing.* www.R-project.org/. Accessed Jan 2017.

10. Sexton J.O., Song X.-P., Feng M., Noojipady P., Anand A., Huang C., Kim D.-H., Collins K.M., Channan S., DiMiceli C., et al. 2013 Global, 30-m resolution continuous fields of tree cover: Landsat-based rescaling of MODIS vegetation continuous fields with lidar-based estimates of error. *Int J Digital Earth* **6**(5), 427-448. (doi:10.1080/17538947.2013.786146).

11. Jarvis A., Reuter H., Nelson A., Guevara E. 2008 *Hole-filled SRTM for the globe Version 4.* CGIAR. http://srtm.csi.cgiar.org. Accessed June 2013.

12. Hijmans R.J. 2015 *raster: Geographic data analysis and modeling. R package version 2.4-15.* http://CRAN.R-project.org/package=raster. Accessed November 2013.

13. Hijmans R.J., Cameron S.E., Parra J.L., Jones P.G., Jarvis A. 2005 Very high resolution interpolated climate surfaces for global land areas. *Int J Climatol* **25**(15), 1965-1978. (doi:10.1002/joc.1276).

14. Trabucco A., Zomer R.J., Bossio D.A., van Straaten O., Verchot L.V. 2008 Climate change mitigation through afforestation/reforestation: A global analysis of hydrologic impacts with four case studies. *Agric, Ecosyst Environ* **126**(1–2), 81-97. (doi:http://dx.doi.org/10.1016/j.agee.2008.01.015).

15. Trabucco A., Zomer R.J. 2010 *Global Soil Water Balance Geospatial Database. CGIAR Consortium for Spatial Information.* Information C.C.f.S. http://www.cgiar-csi.org/data/global-high-resolution-soil-water-balance. Accessed August 2014.

16. New M., Lister D., Hulme M., Makin I. 2002 A high-resolution data set of surface climate over global land areas. *Clim Res* **21**(1), 1-25.

17. Nachtergaele F., Batjes N. 2012 *Harmonized world soil database*. Rome, Food and Agriculture Organization.

18. Nordpil. 2010 *World database of large urban areas, 1950-2050. Vers. 1.1.* https://nordpil.com/resources/world-database-of-large-cities/. Accessed July 2014.

19. IUCN, UNEP. 2009 *The World Database on Protected Areas (WDPA).* World Conservation Monitoring Centre. http://www.wdpa.org/. Accessed August 2014.

20. Balk D.L., Deichmann U., Yetman G., Pozzi F., Hay S.I., Nelson A. 2006 Determining Global Population Distribution: Methods, Applications and Data. *Adv Parasitol* **62**, 119-156.

21. Center for International Earth Science Information Network, International Food Policy Research Institute, The World Bank, Centro Internacional de Agricultura Tropical. 2011 Global Rural-Urban Mapping Project, Version 1 (GRUMPv1): Population Density Grid. (Palisades, NY, NASA Socioeconomic Data and Applications Center.

22. FAO. 2008 *Global ecofloristic zones. Adapted by Ruesch, Aaron, and Holly K. Gibbs.* Food and Agricultural Organization of the United Nations. http://databasin.org/datasets/dc4f6efd1fa84ea99df61ae9c5b3b763. Accessed March 2015.

23. Ramankutty N., Foley J.A., Norman J., McSweeney K. 2002 The global distribution of cultivable lands: current patterns and sensitivity to possible climate change. *Global Ecol Biogeogr* **11**(5), 377-392. (doi:10.1046/j.1466-822x.2002.00294.x).

24. Hansen M.C., Potapov P.V., Moore R., Hancher M., Turubanova S.A., Tyukavina A., Thau D., Stehman S.V., Goetz S.J., Loveland T.R., et al. 2013 High-Resolution Global Maps of 21st-Century Forest Cover Change. *Science* **342**(6160), 850-853. (doi:10.1126/science.1244693).

25. Rossiter D.G. 2014 *Technical Note: Statistical methods for accuracy assesment of classified thematic maps.* http://www.css.cornell.edu/faculty/dgr2/teach/R/R_ac.pdf. Accessed January 2016.

26. Liu J., Liu M., Tian H., Zhuang D., Zhang Z., Zhang W., Tang X., Deng X. 2005 Spatial and temporal patterns of China's cropland during 1990–2000: An analysis based on Landsat TM data. *Remote Sens Environ* **98**(4), 442-456. (doi:http://dx.doi.org/10.1016/j.rse.2005.08.012).

27. Shimada M., Itoh T., Motooka T., Watanabe M., Shiraishi T., Thapa R., Lucas R. 2014 New global forest/non-forest maps from ALOS PALSAR data (2007–2010). *Remote Sens Environ* **155**, 13-31. (doi:http://dx.doi.org/10.1016/j.rse.2014.04.014).

28. Chen J., Chen J., Liao A., Cao X., Chen L., Chen X., He C., Han G., Peng S., Lu M., et al. 2015 Global land cover mapping at 30 m resolution: A POK-based operational approach. *ISPRS J Photogramm* **103**, 7-27. (doi:http://dx.doi.org/10.1016/j.isprsjprs.2014.09.002).

29. Franklin J. 2010 *Mapping species distributions: spatial inference and prediction*. Cambridge, Cambridge University Press.

30. Hijmans R.J., Phillips S., Leathwick J., Elith J. 2013 *dismo: Species distribution modeling. R package version 0.8-17.* http://CRAN.R-project.org/package=dismo. Accessed November 2013.

31. Phillips S.J., Dudik M. 2008 Modeling of species distributions with Maxent: new extensions and a comprehensive evaluation. *Ecography* **31**(2), 161-175. (doi:10.1111/j.0906-7590.2008.5203.x).

32. Elith J., Phillips S.J., Hastie T., Dudík M., Chee Y.E., Yates C.J. 2011 A statistical explanation of MaxEnt for ecologists. *Divers Distrib* **17**(1), 43-57.

33. Zhang Y., Song C. 2006 Impacts of Afforestation, Deforestation, and Reforestation on Forest Cover in China from 1949 to 2003. *J For* **104**(7), 383-387.

34. Qin Y., Xiao X., Dong J., Zhang G., Shimada M., Liu J., Li C., Kou W., Moore Iii B. 2015 Forest cover maps of China in 2010 from multiple approaches and data sources: PALSAR, Landsat, MODIS, FRA, and NFI. *ISPRS J Photogramm* **109**, 1-16. (doi:http://dx.doi.org/10.1016/j.isprsjprs.2015.08.010).

35. Potapov P., Yaroshenko A., Turubanova S., Dubinin M., Laestadius L., Thies C., Aksenov D., Egorov A., Yesipova Y., Glushkov I., et al. 2008 Mapping the Worlds Intact Forest Landscapes by Remote Sensing. *Ecol Soc* **13**.

36. Zeng W., Tomppo E., Healey S.P., Gadow K.V. 2015 The national forest inventory in China: history - results - international context. *Forest Ecosystems* **2**(1), 1-16. (doi:10.1186/s40663-015-0047-2).

37. Liu J., Zhang Z., Xu X., Kuang W., Zhou W., Zhang S., Li R., Yan C., Yu D., Wu S., et al. 2010 Spatial patterns and driving forces of land use change in China during the early 21st century. *J Geograph Sci* **20**(4), 483-494. (doi:10.1007/s11442-010-0483-4).

38. FAO. 2010 Global Forest Resources Assessment 2010. In *FAO Forestry Paper 163* (ed. Food and Agriculture Organization). Rome.

39. FAO. 2000 Global Forest Resources Assessment 2000. (ed. Food and Agriculture Organization). Rome, Food and Agriculture Organization of the United Nations.

40. Friedl M.A., Sulla-Menashe D., Tan B., Schneider A., Ramankutty N., Sibley A., Huang X. 2010 MODIS Collection 5 global land cover: Algorithm refinements and characterization of new datasets. *Remote Sens Environ* **114**(1), 168-182. (doi:http://dx.doi.org/10.1016/j.rse.2009.08.016).

41. Bontemps S., Defourny P., VB E., Arino O., Kalogirou V., Perez J.R. 2011 GlobCOVER 2009 products description and validation report. (ESA)

44. Greenpeace, University of Maryland, World Resources Institute, Transparent World. 2016 *Intact Forest Landscapes 2000/2013* www.globalforestwatch.org. Accessed February 2016.
